# Genetic Variability in Cisplatin Metabolism in Kidney Injury in Patients With Head and Neck Squamous Cell Carcinoma Undergoing Definitive Chemoradiotherapy

**DOI:** 10.1002/hed.28179

**Published:** 2025-05-08

**Authors:** Ericka Francislaine Dias Costa, Ana Maria Castro Ferreira, Marilda Mazzali, Gustavo Jacob Lourenço, Carmen Silvia Passos Lima

**Affiliations:** ^1^ Laboratory of Cancer Genetics, School of Medical Sciences University of Campinas São Paulo Brazil; ^2^ Department of Internal Medicine, School of Medical Sciences University of Campinas São Paulo Brazil; ^3^ Department of Radiology and Oncology, School of Medical Sciences University of Campinas São Paulo Brazil

**Keywords:** cisplatin, head and neck squamous cell carcinoma, nephrotoxicity, pharmacogenomics, single nucleotide variants

## Abstract

**Background:**

This study investigated the roles of single nucleotide variants (SNVs) in genes of CDDP metabolism and their association with kidney dysfunction in patients with head and neck squamous cell carcinoma (HNSCC).

**Methods:**

A total of 109 patients with locally advanced HNSCC, treated with CDDP, had renal function evaluated by serum creatinine level and CKD‐EPI formula, and underwent genotyping by polymerase chain reaction.

**Results:**

Patients with *GSTT1* present and *ERCC1* c.354CT or TT genotypes showed 4.94% and 8.94% renal function reduction, respectively. *GSTT1* present with *TP53* c.215G>C (17.67%), *GSTP1* c.313A>G with *ERCC1* c.354C>T (17.57%), *GSTP1* c.313A>G with *MLH1* c.93G>A (12.49%), *GSTP1* c.313A>G with *MSH3* c.3133A>G (12.19%), *ERCC1* c.354C>T with *MLH1* c.93G>A (18.85%) and *ERCC1* c.354C>T with *MSH3* c.3133A>G (13.38%) combined genotypes were also associated with substantial declines in renal function.

**Conclusions:**

Our data suggest that isolated and combined SNVs in genes enrolled in CDDP metabolism can be used to select patients for treatments that spare the kidneys from adverse effects.

## 1 Introduction

Head and neck squamous cell carcinoma (HNSCC) is a common malignancy worldwide, accounting for approximately 4.6% of global cancer deaths [1], and patients with locally advanced HNSCC are usually treated with cisplatin (CDDP)‐based chemoradiotherapy [2].

**FIGURE 1:**
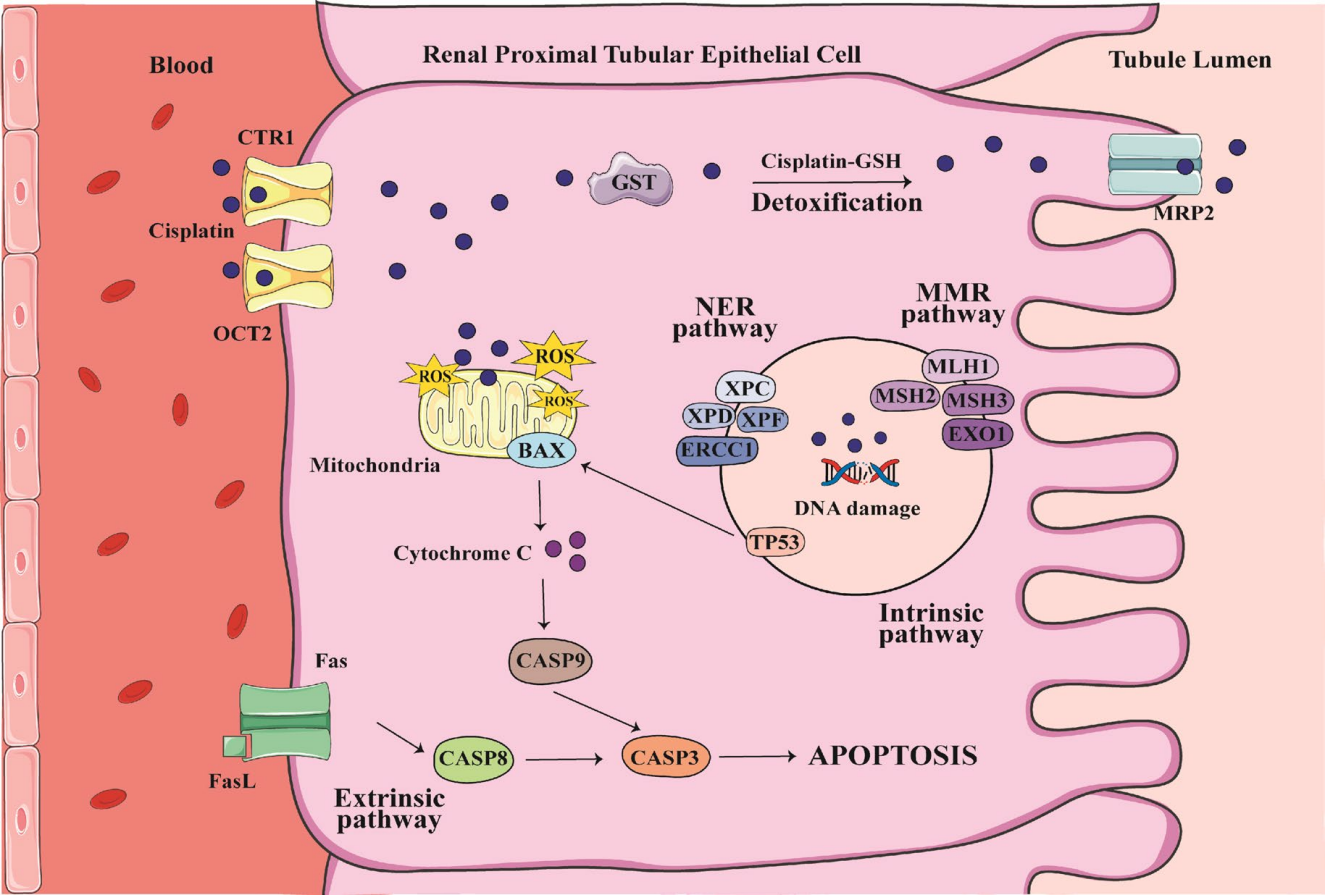
Cisplatin is transported into renal proximal tubular epithelial cells via the copper transporter 1 (CTR1) and the organic cation transporter 2 (OCT2). Once internalized, a fraction of cisplatin undergoes detoxification through conjugation with glutathione (GSH), a reaction catalyzed by glutathione S‐transferase (GST), followed by efflux via multidrug resistance‐associated protein 2 (MRP2). However, the remaining cisplatin forms covalent DNA adducts, leading to replication stress, transcriptional inhibition, and genomic instability. To mitigate this genotoxic insult, cells activate DNA repair pathways, including nucleotide excision repair (NER)‐mediated by proteins such as XPC, XPD, XPF, and ERCC1 and mismatch repair (MMR), involving MLH1, MSH2, MSH3, and EXO1. In parallel, cisplatin promotes oxidative stress by inducing the accumulation of reactive oxygen species (ROS), which cause mitochondrial dysfunction and activate the pro‐apoptotic protein BAX. When DNA damage is beyond repair, tumor protein p53 (TP53) orchestrates apoptosis via the intrinsic (mitochondrial) pathway, characterized by cytochrome C release and caspase‐9 (CASP9) activation. Additionally, the extrinsic apoptotic pathway is engaged through Fas ligand (FasL) binding to its receptor, leading to caspase‐8 (CASP8) activation. Both apoptotic cascades converge on caspase‐3 (CASP3), executing programmed cell death and contributing to cisplatin‐induced nephrotoxicity. [Color figure can be viewed at wileyonlinelibrary.com]

CDDP exerts its effects primarily by binding to DNA, triggering damage that activates multiple cellular responses [3]. These responses include detoxification, DNA repair, and programmed cell death. Detoxification involves the binding of CDDP to glutathione, a reaction mediated by glutathione S‐transferase (GST) enzymes encoded by genes like *GSTM1*, *GSTT1*, and *GSTP1* [4, 5]. DNA binding by CDDP generates adducts and reactive oxygen species (ROS), which stimulate repair pathways. Proteins encoded by genes such as *XPC*, *XPD*, *XPF*, *ERCC1*, *MLH1*, *MSH2*, *MSH3*, and *EXO1* play critical roles in nucleotide excision repair (NER) and mismatch repair (MMR), facilitating the resolution of CDDP‐induced DNA damage [3, 4, 5, 6, 7, 8]. When repair mechanisms fail, apoptotic pathways are triggered. This involves key regulators such as *TP53*, *CASP3*, *FAS*, and *FASL*, which lead to the elimination of cells harboring irreparable damage [5, 9] (Figure 1).

Despite unequivocal beneficial effects, CDDP use is limited by its side effects [10, 11, 12], including nephrotoxicity [7]. The CDDP‐induced acute kidney injury (AKI) results from its accumulation in renal cells, causing a decline in vascular, glomerular, and tubular functions [13]. Inherited variability in CDDP‐induced nephrotoxicity risk has been linked to single nucleotide variants (SNVs) in genes involved in CDDP metabolism. Previous studies conducted by our group identified associations between *XPD* c.2251A>C and c.934G>A, and *ERCC1* c.354C>T [14], *MSH3* c.3133G>A and *EXO1* c.1762G>A [15], *GSTT1* and *GSTP1* c.313A>G [16] with nephrotoxicity outcomes. Associations of *ERCC1* c.354C>T [17, 18, 19, 20], *TP53* c.215G>C [20] and *GSTT1* [19] with AKI have also been reported. Additionally, other SNVs have also been reported to exhibit associations with platinum‐induced nephrotoxicity (Table S1).

It is worth mentioning that renal function was previously assessed using the chromium‐51 labeled ethylenediamine tetraacetic acid (^51^Cr‐EDTA) method [14, 15, 16], serum creatinine (Scr) level [20], Cockcroft‐Gault formula [17, 19], and the formula suggested by Stevens et al. [18, 21].

Even though ^51^Cr‐EDTA has been considered the gold standard for evaluating glomerular filtration rate (GFR) [22], this method is not routinely available due to its cost and complexity [23]. To address this limitation, the current study incorporates the Chronic Kidney Disease Epidemiology Collaboration (CKD‐EPI) formula, a more feasible accepted method for estimating GFR (eGFR) from Scr [24], to analyze associations of SNVs in genes of CDDP pathways with nephrotoxicity in almost the same patient cohort analyzed in previous studies conducted by our group. Furthermore, since only SNVs in genes of isolated pathways of CDDP metabolism were previously analyzed, the current study also aims to evaluate whether combinations of inherited defects may induce more nephrotoxicity than isolated ones.

## 2 Materials and Methods

### 2.1 Patients and Clinicopathological Aspects

This cohort prospectively enrolled 109 HNSCC patients diagnosed at the Clinical Oncology Service of the General Hospital of the University of Campinas between June 2011 and February 2014.

Patients eligible for the study were those with a GFR exceeding 45 mL/min who received CDDP chemoradiation as a definitive treatment. This treatment was indicated for individuals with unresectable tumors, those who declined surgery due to anticipated complications, or as part of an organ preservation protocol. Exclusions were made for patients undergoing induction, adjuvant, or palliative therapies.

Data collected from medical records included age at diagnosis, gender, tobacco and alcohol use, histological tumor grade, body mass index (BMI), and the presence of diabetes and systemic hypertension. Based on established criteria, patients were categorized as smokers [25], drinkers [25], hypertensive [26], and diabetic [27]. Tumor diagnoses adhered to World Health Organization standards [28] and staging was conducted according to the American Joint Committee on Cancer guidelines [29].

The research was carried out in accordance with the Declaration of Helsinki and received ethical approval from the Ethics Committees of the University of Campinas (Protocol n° 274/2011; CAAE: 0218.0.146.000–11). All participants provided written informed consent before joining the study.

### 2.2 Treatment

Patients were treated with high‐dose CDDP (80–100 mg/m^2^), administered on days 1, 22, and 43, in combination with single daily fractionated radiotherapy (70 Gy, delivered at 2 Gy/day) [12]. Patients who did not achieve a satisfactory response or experienced tumor recurrence underwent surgical resection when eligible. For those unable to undergo surgery, intravenous methotrexate was provided as a palliative care option [30].

### 2.3 Kidney Function

Renal function was assessed by calculating eGFR using the 2021 CKD‐EPI equation, before treatment initiation and 30 days post‐treatment. eGFR was calculated using Scr levels, gender, and age, according to the 2021 CKD‐EPI equation [24]. eGFR = 142 × min (standardized Scr/K,1) ^α^ * max (standardized Scr/K,1) ^−1.200^ * 0.9938 ^age in years^ * 1.012 [if female], where: Scr = serum creatinine in mg/dL; K = 0.7 (females) or 0.9 (males); *α* = −0.241 (females) or −0.302 (males); min (standardized Scr/K,1) = the minimum of Scr/K or 1; max (standardized Scr/K,1) = the maximum of Scr/K or 1. Creatinine levels were measured using the automated Hitachi‐Roche system, employing the Jaffé method [31]. eGFR was normalized to a surface area of mL/min/1.73m^2^.

The percentual variation in kidney function was calculated by determining the difference between baseline (pre‐chemotherapy) and follow‐up (post‐chemotherapy) values for eGFR divided by the pre‐chemotherapy value and represented as ΔeGFR. The values of ΔeGFR that were positive indicated a percentage decline in renal function, while negative values were associated with an improvement in renal function.

### 2.4 Genotyping

Genotyping was conducted on DNA extracted from peripheral blood samples of patients. The following methods were employed: multiplex polymerase chain reaction (PCR) for *GSTM1* and *GSTT1* [32]; PCR followed by enzymatic digestion for *GSTP1* c.313A>G (rs1695) [33], *XPC* c.2815A>C (rs2228001) [34], *XPD* c.934G>A (rs1799793) and c.2251A>C (rs13181) [35], *XPF* c.2505 T>C (rs1799801) [36], *ERCC1* c.354C>T (rs11615) [37], *MLH1* c.‐93G>A (rs1800734) [38], *MSH2* c.211 + 9C>G (rs2303426) [39], *EXO1* c.1765G>A (rs1047840) [40], *TP53* c.215G>C (rs1042522) [41], *CASP3* c.‐1191A>G (rs12108497) [42] and c.‐182‐247G>T (rs4647601) [43], *FAS* c.‐1378G>A (rs2234767) [44] and c.‐671A>G (rs1800682) [45], and *FASL* c.‐844C>T (rs763110) [44]. Additionally, real‐time PCR was performed for *MSH3* c.3133G>A (rs26279) [46]. All reactions included positive and negative controls. To ensure accuracy, 10% of the genotyping results were reanalyzed in independent experiments, and full concordance was observed.

### 2.5 Statistical Analysis

The study evaluated all clinicopathological factors as well as individual and combined genotypes in relation to renal function. For each SNV, individuals were classified into three possible genotypes: homozygous for the ancestral allele, heterozygous, or homozygous for the variant allele. The functional grouping was based on the known or predicted impact of each allele on protein function, particularly regarding detoxification, DNA repair, and apoptosis mechanisms [35, 42, 43, 47, 48, 49, 50, 51, 52, 53, 54, 55, 56, 57, 58, 59, 60] as presented in Table S2. Genotypes were grouped according to the functional effect of the alleles: individuals carrying homozygous genotypes associated with normal protein function were classified into Group 1, while those with homozygous genotypes associated with reduced function were classified into Group 2. Due to the uncertainty surrounding the functional role of heterozygous genotypes, which carry both alleles, two complementary analyses were conducted: In the first, heterozygous individuals were grouped with those presenting preserved function to assess whether the presence of one functional allele might be sufficient to maintain normal activity. In the second, heterozygous individuals were grouped with reduced‐function genotypes to test whether a single variant allele could already impact biological function.

Univariate analyses were conducted using the *T*‐test, including only variables with *p‐*values less than 0.10 and more than 10 individuals in each group for subsequent multivariate analyses. Multiple linear regression was employed to assess the impact of various factors on renal function decline. Pearson's correlation coefficient was used to evaluate the relationship between cumulative CDDP and ΔeGFR, with the purpose of verifying if it could be included in multivariate analysis. All results are presented as correlation coefficients (*r*). Statistical analyses were performed using RStudio (R Core Team, USA), with significance defined as *p‐*values ≤ 0.05.

## 3 Results

### 3.1 Clinicopathological Aspects of Patients

The study cohort had a mean age of 56 years, with the majority being male and smokers. Most patients were alcohol drinkers, nonhypertensive, and with no history of diabetes. Tumor location was predominantly in the pharynx, followed by the larynx and oral cavity. The majority of tumors were well‐ or moderately differentiated, and most patients presented with advanced tumor stages. While the median BMI fell within the normal range, most patients were classified as underweight. The median CDDP dose was 418.2 mg. eGFR decreased from 99.8 mL/min before treatment to 95.0 mL/min post‐treatment, reflecting a 3.6% reduction (Table 1).

**TABLE 1:** Clinicopathological aspects and kidney function before and after treatment with cisplatin chemoradiation in patients with head and neck squamous cell carcinoma.

Variable	Mean (SD) or *N* (%)
Age at diagnosis	56 (± 8.6)
Sex	
Male	101 (92.7)
Female	8 (7.3)
Tobacco consumption	
Smokers	106 (97.2)
Non‐smokers	3 (2.28)
Alcohol consumption	
Drinkers	100 (91.7)
Abstainers	9 (8.3)
Hypertension	
Yes	28 (25.7)
No	81 (74.3)
Diabetes	
Yes	12 (11.0)
No	97 (89.0)
Tumor location	
Oral cavity	13 (11.9%)
Pharynx	68 (62.4%)
Larynx	28 (25.7%)
Histological grade^a^	
Well or moderately differentiated	73 (67.0)
Poorly or undifferentiated	16 (14.7)
T stage	
T1	7 (6.4)
T2	20 (18.3)
T3	30 (27.6)
T4	52 (47.7)
N stage	
N0	20 (18.3)
N1	22 (20.2)
N2	55 (50.5)
N3	12 (11.0)
M stage	
M0	109 (100.0)
M1	0 (0.0)
Tumor stage	
I or II	6 (5.5)
III or IV	103 (94.5)
Body mass index (kg/m^2^)	19.6 ± 4.05
Underweight (< 20)	67 (61.5%)
Normal weight (20–24.9)	28 (25.7%)
Overweight (≥ 25)	14 (12.8%)
Cumulative dose of cisplatin (mg)	418.2 ± 93.3
eGFR (CKD‐EPI)^b^	
Before	99.8 ± 15.6
After	95.0 ± 18.7
Δ (%)	3.6 ± 19.8

*Note:* Data presented as number (*N*) or mean ± standard deviation (SD).

^a^ The number of patients differed from the total quoted in the study because it was not possible to obtain consistent information in some cases. eGFR: glomerular filtration rate estimated. ΔeGFR (%) represents pre‐chemotherapy—post chemotherapy values divided by the pre‐chemotherapy value.

^b^ *p*‐value = 0.005.

### 3.2 Kidney Injury After CDDP Treatment

The results of univariate analysis of associations of clinicopathological aspects and genotypes of isolated and combined SNVs with renal changes are presented in Table S3, and all factors with potential roles in renal function decline (*p* < 0.10) are presented in Table 2. Patients with *GSTP1* c.313AG or GG combined with *ERCC1* c.354CT or TT (9.38% vs. −7.78%), *MLH1* c.93GA or AA (13.33% vs. 1.26%) and *MSH3* c.3133AG or GG (9.07% vs. −2.81%) exhibited a decline in renal function compared to those with the remaining genotypes. Patients with the *GSTT1* present and *TP53* c.215CC combined genotype showed a mean decline of 8.86% in renal function after CDDP treatment when compared to those with the *GSTT1* null plus *TP53* c.215GC or GG genotype. Patients with the *ERCC1* c.354CT or TT and *MLH1* c.93GA or AA combined genotype exhibited a decline in renal function of 4.34% after CDDP treatment compared to those with other genotypes (−13.09%). Clinicopathological aspects and genotypes of isolated SNVs were not associated with changes in renal functional changes.

**TABLE 2:** Univariate analysis of age and single nucleotide variants in renal function of patients with head and neck squamous cell carcinoma after cisplatin treatment.

Variable	*N*	∆eGFR (%)	*p*‐value
		(Mean ± SD)	
Age			
≤ 56 years	51	7.11 ± 19.63	0.08
> 56 years	58	0.42 ± 19.60	
*Isolated SNVs in detoxification genes*			
*GSTT1*			
Present	92	5.39 ± 18.42	0.07
Null	17	−6.39 ± 24.37	
*GSTP1* c.313A>G			
AA	54	−0.05 ± 21.43	0.06
AG or GG	55	7.09 ± 17.57	
*Isolated SNV in NER repair gene*			
*ERCC1* c.354C>T			
CC	28	−3.09 ± 25.03	0.09
CT or TT	81	5.85 ± 17.26	
*Combined SNVs in detoxification genes*			
*GSTM1* + *GSTP1* c.313A>G			
Present + AA	21	−7.73 ± 26.80	0.08
Null + AG or GG	28	4.48 ± 18.71	
*Combined SNVs in detoxification and NER/MMR repair genes*			
*GSTP1* c.313A>G *+ XPC* c.2815A>C			
AA + AA	21	−2.04 ± 22.52	0.07
AG or GG + AC or CC	35	8.83 ± 18.15	
*GSTP1* c.313A>G *+ ERCC1* c.354C>T			
AA + CC	13	−7.78 ± 28.08	**0.05**
AG or GG + CT or TT	40	9.38 ± 15.18	
*GSTP1* c.313A>G *+ MLH1* c.93G>A			
AA + GG	30	1.26 ± 21.81	**0.02**
AG or GG + GA or AA	22	13.33 ± 15.83	
*GSTP1* c.313A>G *+ MSH2* c.211 + 9C>G			
AA + CC	14	−5.96 ± 23.29	0.06
AG or GG + CG or GG	44	7.37 ± 15.60	
*GSTP1* c.313A>G *+ MSH3* c.3133A>G			
AA + AA	27	−2.81 ± 22.05	**0.03**
AG or GG + AG or GG	21	9.07 ± 15.80	
*Combined SNVs in detoxification and intrinsic/extrinsic apoptosis genes*			
*GSTT1 + TP53* c.215G>C			
Present + CC	10	8.86 ± 14.70	**0.02**
Null + GC or GG	16	−9.38 ± 21.71	
*GSTP1* c.313A>G *+ FASL* c.‐844C>T			
AA + TT	16	−6.60 ± 23.60	0.06
AG or GG + CT or CC	44	6.47 ± 18.30	
*Combined SNVs in NER/MMR repair genes*			
*XPD* c.2251A>C *+ EXO1* c.1765G>A			
AA + GG	20	8.02 ± 15.97	0.09
AC or CC + GA or AA	29	−2.07 ± 24.22	
*ERCC1* c.354C>T *+ MLH1* c.93G>A			
CC + GG	15	−13.09 ± 24.13	**0.02**
CT or TT + GA or AA	33	4.34 ± 19.83	
*ERCC1* c.354C>T *+ MSH3* c.3133A>G			
CC + AA	16	−6.07 ± 25.42	0.07
CT or TT + AG or GG	36	7.03 ± 16.50	

*Note:* Results with significant *p‐*values (≤ 0.05) are presented in bold letters. The number of patients in each genotype combination may vary because only individuals with both SNVs available were included in the analysis. *N*: number of patients. eGFR: estimated glomerular filtration rate. ΔeGFR (%) represents pre‐chemotherapy–post chemotherapy values, divided by the pre‐chemotherapy value. NER: nucleotide excision repair pathway. MMR: mismatch repair pathway.

The cumulative CDDP dose was positively correlated with the decline in renal function, with a correlation coefficient (*r*) of 0.28 (*p* = 0.003).

The results of multivariate analyses of associations of factors with potential roles (*p* < 0.10) or positively correlated with renal function decline are presented in Table 3. Patients who received high cumulative CDDP doses presented a decline in renal function after CDDP treatment, with an estimated reduction of 0.04%. Patients with the *GSTT1* present and *ERCC1* c.354CT or TT isolated genotypes presented a decline in kidney function of 4.94% and 8.94%, respectively. Patients with the *GSTP1* c.313AG or GG and *ERCC1* c.354CT or TTC>T, *MLH1* c.93GA or A, or *MSH3* c.3133AG or GG combined genotypes showed up to a 17.57% reduction in renal function after CDDP treatment. A decline of 17.67% in renal function post‐CDDP treatment was observed in patients with the *GSTT1* present combined with the *TP53* c.215CC genotype. Renal function declines of 18.85% and 13.38% were observed in patients with *ERCC1* c.354CT or TT and *MLH1* c.93GA or AA or *MSH3* c.3133AG or GG combined genotypes, respectively (Table 3).

**TABLE 3:** Multiple linear regression analysis of clinicopathological aspects and single nucleotide variants in renal function of patients with head and neck squamous cell carcinoma after cisplatin treatment.

Variable	Estimate	95% CI	*p*‐value
Age (≤ 56 years)	−4.48	−11.73 to 2.77	0.22
Cisplatin dose (mg)	−0.04	−0.09 to −0.01	**0.01**
*Isolated SNVs in detoxification genes*			
*GSTT1* (present)	−4.94	−9.80 to −0.09	**0.05**
*GSTP1* c.313A>G (AG or GG)	−6.42	−13.42 to 0.58	0.07
*Isolated SNV in NER repair gene*			
*ERCC1* c.354C>T (CT or TT)	−8.94	−16.93 to −0.94	**0.03**
*Combined SNVs in detoxification genes*			
*GSTM1 + GSTP1* c.313A>G (Null + AG or GG)	−12.21	−25.29 to 0.86	0.07
*Combined SNVs in detoxification and NER/MMR repair genes*			
*GSTP1* c.313A>G *+ XPC* c.2815A>C (AG or GG + AC or CC)	−10.88	−21.88 to 0.13	0.05
*GSTP1* c.313A>G *+ ERCC1* c.354C>T (AG or GG + CT or TT)	−17.57	−29.39 to −5.75	**0.004**
*GSTP1* c.313A>G *+ MLH1* c.93G>A (AG or GG + GA or AA)	−12.49	−23.30 to −1.67	**0.02**
*GSTP1* c.313A>G *+ MSH2* c.211 + 9C>G (AG or GG + CG or GG)	−9.44	−20.42 to 1.54	0.09
*GSTP1* c.313A>G *+ MSH3* c.3133A>G (AG or GG + AG or GG)	−12.19	−23.50 to −0.89	**0.04**
*Combined SNVs in detoxification and intrinsic/extrinsic apoptosis genes*			
*GSTT1 + TP53* c.215G>C (Present + CC)	−17.67	−33.41 to −1.93	**0.03**
*GSTP1* c.313A>G *+ FASL* c.‐844C>T (AG or GG + CT or CC)	−10.81	−22.37 to 0.74	0.06
*Combined SNVs in NER/MMR repair genes*			
*XPD* c.2251A>C *+ EXO1* c.1765G>A (AA + GG)	−10.09	−22.53 to 2.35	0.11
*ERCC1* c.354C>T *+ MLH1* c.93G>A (CT or TT + GA or AA)	−18.85	−31.90 to −5.81	**0.006**
*ERCC1* c.354C>T *+ MSH3* c.3133A>G (CT or TT + AG or GG)	−13.38	−25.11 to −1.65	**0.03**

*Note:* Changes in renal function were evaluated by ΔeGFR (%), where eGFR was estimated glomerular filtration rate, and ΔeGFR (%) represents pre‐chemotherapy—post chemotherapy values, divided by pre‐chemotherapy value.

Abbreviations: CI: confidence interval, MMR: mismatch repair pathway, NER: nucleotide excision repair pathway.

## 4 Discussion

This prospective study, we assessed the influence of SNVs associated with intracellular detoxification, DNA repair, and apoptosis in CDDP‐induced nephrotoxicity using the CKD‐EPI (2021) formula. While the ^51^Cr‐EDTA clearance method is the gold standard for measuring GFR [61, 62], its application in routine clinical practice is hindered by its cost, invasiveness, use of radioactivity, and limited availability [63]. CKD‐EPI is an alternative to ^51^Cr‐EDTA clearance due to its lower cost, ease of use, and broader applicability, as demonstrated in studies focusing on CDDP‐induced nephrotoxicity [61, 64, 65, 66].

In our study, the eGFR calculated using the CKD‐EPI formula decreased from 99.8 to 95.0 post‐treatment, reflecting a modest reduction of 3.6%. Lindberg et al. [61] and Stormoen et al. [66] observed similar reductions of 4.9% and 7.1% in HNSCC patients treated with CDDP and urothelial carcinoma patients treated with CDDP or carboplatin, respectively. De Godoy Torso et al. [65] reported a 7% decline in eGFR measured 20 days after the first dose of CDDP in HNSCC patients. Lastly, Funakoshi et al. [64] observed an 8.2% eGFR reduction in cancer patients treated with CDDP‐based therapy.

The mechanisms of CDDP‐induced AKI are complex, including nuclear and mitochondrial damage, apoptosis activation, oxidative stress, and inflammation [9]. This nephrotoxicity primarily arises from CDDP accumulation in renal tubular cells, resulting in direct tubular proximal epithelial toxicity [13, 67].

We observed a positive correlation of cumulative CDDP dose with decline in renal function, and cumulative CDDP dose was also seen as an independent factor for renal decline after chemoradiation. High‐dose CDDP was an independent risk factor for renal damage identified by increases in Scr levels [68, 69] and reduced eGFR calculated by the Modification of Diet in Renal Disease (MDRD) [70], Cockcroft‐Gault [71] and Stevens et al. [18, 21] formulas. In a systematic review, Kooijmans et al. [72] found the cumulative dose of CDDP as a risk factor for nephrotoxicity, independent of the method utilized to calculate renal function, Scr, eGFR (CKD‐EPI, MDRD, Cockcroft‐Gault formula), and measured GFR (inulin clearance).

Genetic factors involved in CDDP metabolism influenced the severity of AKI in our study. In this context, we observed that HNSCC patients with *GSTT1* present genotype had a decline of almost 5% in renal function, and the reduction in eGFR was of approximately 18% in patients with *GSTT1* present and *TP53* c.215CC combined genotype. Supporting our results, a preliminary analysis of this cohort conducted by our group reported that patients harboring the *GSTT1* showed a more significant reduction in ^51^Cr‐EDTA GFR after chemoradiation than those with *GSTT1* null genotype [16]. In contrast, Khrunin et al. [19] and Liu et al. [20] have indicated that *GSTT1* null and *TP53* c.215GG genotype were linked to an increased risk of platinum‐induced nephrotoxicity, respectively. The discrepancy between our findings and previous results is not easy to explain and could be attributed to variations in sample size. Our study included 109 patients, whereas Khrunin et al. [19] analyzed 87, and Liu et al. [20] included 41, which may have influenced the outcomes. In fact, GSTT1 is responsible for catalyzing the conjugation of toxic substances with glutathione (GSH), facilitating their detoxification and excretion [48], and in renal proximal tubular cells, CDDP is biotransformed into nephrotoxic metabolites through the formation of GSH‐CDDP conjugates, which accumulate in the renal tubular epithelial inducing cell death [73]. Furthermore, the senescence and telomere shortening of renal tubular epithelial cells constitutes a critical cellular event for the progression of AKI [74]. It was recently reported that CDDP is associated with senescence and telomere shortening in gastric cancer cells, as evidenced by increased TP53 expression and reduced telomerase activity following treatment with CDDP and resveratrol [75]. Similarly, CDDP treatment induced premature renal senescence in a murine model, mediated by TP53 [76]. To the best of our knowledge, the roles of TP53 in renal cellular senescence and in HNSCC are still unknown. Thus, we hypothesized that our patients with *GSTT1* present had accumulation of GSH‐CDDP conjugates in renal tubular epithelial cells, having AKT as a consequence, and the combination of reduced apoptotic capacity conferred by the *TP53* c.215CC genotype [57] exacerbated renal injury by promoting cellular senescence and telomere shortening. Functional studies need to be performed to confirm our hypothesis.

Our patients with the *ERCC1* c.354CT or TT genotype had a decline of almost 9% in renal function after CDDP chemoradiation. In fact, a tendency for association of the *ERCC1* c.354TT genotype with renal toxicity (*p*‐value = 0.06), using ^51^Cr‐EDTA, had already been identified in a preliminary analysis of this cohort of patients (*n* = 90) [14]. Corroborating our findings, Tzvetkov et al. [18] reported that the T allele was linked to nephrotoxicity in cancer patients receiving CDDP‐containing chemotherapy, and Khrunin et al. [17] and Khrunin et al. [19] observed that the heterozygous genotype increased the risk of nephrotoxicity in ovarian cancer patients undergoing a CDDP‐cyclophosphamide regimen. In contrast, Liu et al. [20] found that the CC genotype was associated with renal function decline in lung cancer patients after platinum‐based treatment. As mentioned previously, the difference between our findings and Liu's study may be due to the smaller number of patients in their analysis. Significant differences were only observed after stratifying into a smaller group of 27 from the original cohort of 104 patients, compared to 109 in our study. This variation in cohort size could have influenced the results. Moreover, reductions in eGFR of approximately 19% and 14% were seen in patients with the *ERCC1* c.354CT or TT and *MLH1* c.93GA or AA and *MSH3* c.3133AG or GG genotypes. These findings indicate that combined defects in CDDP metabolism potentiate renal cell injury. The association of the *MSH3* c.3133GG genotype with nephrotoxicity was seen in a preliminary analysis of this cohort of patients (*n* = 90) following chemoradiation with CDDP [15]. To the best of our knowledge, there are no previous studies focusing on the role of the isolated or combined effect of these SNVs on CDDP‐induced nephrotoxicity. Since the T allele of *ERCC1* c.354C>T [52], A of *MLH1* c.93G>A [53] and G of *MSH3* c.3133A>G [55] were associated with lower DNA repair capacity, the association of these genotypes with CDDP‐induced nephrotoxicity in HNSCC patients is biologically plausible. DNA damage in the renal proximal tubules is an initial trigger induced by CDDP administration [73]. If the damage is mild, DNA repair mechanisms are activated, preventing cell death. However, when the damage is extensive and exceeds the repair capacity, apoptotic pathways are triggered, leading to tubular cell death [73]. Thus, we hypothesize that the reduced DNA repair capacity in renal tubular epithelial cells of individuals carrying the variant alleles of *ERCC1* c.354C>T, *MLH1* c.93G>A, and *MSH3* c.3133A>G promotes apoptosis, leading to tubular cell death and renal injury.

We also observed that patients with *GSTP1* c.313AG or GG genotypes grouped with *ERCC1* c.354CT or TT, *MLH1* c.93GA or AA, and *MSH3* c.3133AG or GG experienced declines of 18%, 13%, and 12% in renal function when compared to patients with the remaining genotypes. A preliminary analysis of this cohort of patients (*n* = 90) analyzed by our group showed that individuals with the *GSTP1* c.313AG or GG genotype experienced a more pronounced reduction in ^51^Cr‐EDTA GFR after chemoradiation compared to those with other genotypes [16]. In addition to forming CDDP‐DNA adducts that trigger cell death, CDDP toxicity is also manifested through the induction of oxidative stress. Under normal physiological conditions, the levels of intracellular ROS are regulated and neutralized by detoxification systems, including GST [77]. Besides, loss of *GSTP1* expression in prostate cancer cells led to increased ROS levels and DNA damage [78]. Elevated oxidative stress has also been observed in end‐stage renal disease patients with the *GSTP1* c.313GG genotype [79]. Thus, we believe that the subgroup of patients with low activity of detoxification and reduced DNA repair may exhibit higher susceptibility to CDDP‐induced nephrotoxicity.

These findings reinforce the potential of pharmacogenetics to guide more personalized CDDP‐based treatments. In clinical practice, genotyping patients for these SNVs may enable risk stratification for nephrotoxicity, allowing dose adjustments and the implementation of nephroprotective strategies in high‐risk individuals, while permitting safer dose escalation in low‐risk patients [18, 80]. Additionally, the integration of genetic information with clinical variables may improve pre‐treatment risk assessments and support decision‐making regarding alternative therapies or future inclusion in clinical trials evaluating platinum analogs with reduced toxicity profiles [81].

In summary, our data showed that *GSTT1* and *ERCC1* c.354C>T SNVs, independently and in combination with other SNVs (*TP53* c.215G>C, *GSTP1* c.313A>G, *MLH1* c.93G>A, and *MSH3* c.3133A>G), act as independent factors for CDDP‐induced nephrotoxicity in HNSCC patients treated with CDDP chemoradiation through their impact on detoxification capacity, DNA repair, and apoptosis efficiency. Our data also indicate eGFR, using Scr levels and 2021 CKD‐EPI equation, as a good method for identification of declines in renal function after CDDP treatment, and as expected, also indicate that combinations of genotypes increase substantially the declines in renal function more than isolated ones. Although a significant number of patients were included in this complex and prospective study, we acknowledge that a larger patient cohort is needed to confirm the roles of inherited variations in different pathways of CDDP metabolism in AKI among HNSCC patients treated with CDDP‐based chemoradiation. If so, they can be used to select HNSCC for individualized chemotherapy regimens.

## Author Contributions

E.F.D.C. and A.M.C.F. participated in statistical analysis, manuscript writing, and revision. M.M. participated in research planning, study design, and revision. G.J.L. participated in statistical analysis and revision. C.S.P.L. participated in research planning, study design, manuscript writing, and revision. All authors have read and agreed to the final version of the manuscript.

## Ethics Statement

The Ethics Committees of the University of Campinas approved this study (register numbers: 274/2011; CAAE: 0218.0.146.000–11).

## Consent

All patients provided informed consent.

## Conflicts of Interest

The authors declare no conflicts of interest.

## Supporting information


Table S1.

Table S2.

Table S3.

## Data Availability

The data that support the findings of this study are available from the corresponding author upon reasonable request.
