# Supplementary material for: Genetic Variability in Cisplatin Metabolism in Kidney Injury in Patients With Head and Neck Squamous Cell Carcinoma Undergoing Definitive Chemoradiotherapy
Source: Head Neck. 2025 May 8;47(10):2683–92. doi: 10.1002/hed.28179 (PMC12434574; doi:10.1002/hed.28179)
Supplement: Supplementary file 1 — Table S1. [file HED-47-2683-s001.doc]

**Supplementary Table S1**. Single nucleotide variants associated with platinum-induced nephrotoxicity.

| **Author(s)** | **Gene** | **rs number** | **Tumor** | **Treatment** | **Method for evaluating nephrotoxicity** |
| --- | --- | --- | --- | --- | --- |
| [80] | *ABCB1* | rs2032582 | Lung cancer | Platinum‐based | Serum creatinine levels/creatinine clearance |
|  | *ABCC1* | rs41410450 |  |  |  |
| [81] | *SLC22A2* | rs316019 | Head and neck, esophageal and non-small-cell lung cancers | Cisplatin-based | CKD-EPI formula |
|  | *ERCC1* | rs3212986 |  |  |  |
|  | *BACH2* | rs4388268 |  |  |  |
| [82] | *SLC22A2* | rs316019 | Neuroblastoma | Cisplatin-based | Serum creatinine levels |
| [83] | *SLC22A2* | rs316019 | Esophageal cancer | Cisplatin-based | Modified MDRD formula |
| [84] | *SLC22A2* | rs316019 | Advanced carcinomas | Cisplatin-based | Equation used for estimating GFR |
| [85] | *SLC22A2* | rs596881 | Genital, head and neck, melanoma, lung, digestive, urinary and other cancers | Cisplatin-based | CKD-EPI formula |
|  | *SLC31A1* | rs12686377 rs7851395 |  |  |  |
| [86] | *EPHX1* | rs1051740 | Ovarian cancer | Cisplatin-based | Cockcroft-Gault formula |
| [87] | *COMT* | rs9332377 | Pulmonary, testicular, head and neck and other cancers | Cisplatin-based | CKD-EPI formula |
| [88] | *ELF3* | rs3740556 | Lung cancer | Platinum‐based | Serum creatinine levels / Creatinine clearance |

CKD-EPI: Chronic Kidney Disease Epidemiology Collaboration equation, MDRD: Modification of Diet in Renal Disease.

**References**

1. Sharma P, Singh N, Sharma S. Genetic variations in ABC transporter genes as a predictive biomarker for toxicity in North Indian lung cancer patients undergoing platinum-based doublet chemotherapy. J Biochem Mol Toxicol 2023;37(3):e23269.
2. Zazuli Z, Otten LS, Drögemöller BI et al. Outcome Definition Influences the Relationship Between Genetic Polymorphisms of ERCC1, ERCC2, SLC22A2 and Cisplatin Nephrotoxicity in Adult Testicular Cancer Patients. Genes (Basel) 2019;10(5):364.
3. Yanagisawa R, Kubota N, Hidaka E et al. Cisplatin-induced nephrotoxicity in patients with advanced neuroblastoma. Pediatr Blood Cancer 2018;65(9):e27253.
4. Hinai Y, Motoyama S, Niioka T, Miura M. Absence of effect of SLC22A2 genotype on cisplatin-induced nephrotoxicity in oesophageal cancer patients receiving cisplatin and 5-fluorouracil: report of results discordant with those of earlier studies. J Clin Pharm Ther. 2013;38(6):498-503.
5. Iwata K, Aizawa K, Kamitsu S et al. Effects of genetic variants in SLC22A2 organic cation transporter 2 and SLC47A1 multidrug and toxin extrusion 1 transporter on cisplatin-induced adverse events. Clin Exp Nephrol. 2012 Dec;16(6):843-51.
6. Chang C, Hu Y, Hogan SL et al. Pharmacogenomic Variants May Influence the Urinary Excretion of Novel Kidney Injury Biomarkers in Patients Receiving Cisplatin. Int J Mol Sci 2017;18(7):1333.
7. Khrunin AV, Khokhrin DV, Moisseev AA, Gorbunova VA, Limborska SA. Pharmacogenomic assessment of cisplatin-based chemotherapy outcomes in ovarian cancer. Pharmacogenomics 2014;15(3):329-337.
8. Agema BC, Koolen SLW, With M et al. Influence of genetic variation in COMT on cisplatin-induced nephrotoxicity in cancer patients. Genes (Basel). 2020;11(4):358.
9. Xu X, Han L, Duan L et al. Association between eIF3α polymorphism and severe toxicity caused by platinum-based chemotherapy in non-small cell lung cancer patients. Br J Clin Pharmacol. 2013;75(2):516-523.
