# Supplementary material for: Genetic Variability in Cisplatin Metabolism in Kidney Injury in Patients With Head and Neck Squamous Cell Carcinoma Undergoing Definitive Chemoradiotherapy
Source: Head Neck. 2025 May 8;47(10):2683–92. doi: 10.1002/hed.28179 (PMC12434574; doi:10.1002/hed.28179)
Supplement: Supplementary file 2 — Table S2. [file HED-47-2683-s003.doc]

**Supplementary Table S2**. Functional roles of wild and variant alleles of single nucleotide variant on genes of cisplatin metabolism

| **SNV** | **Functional analyses** | | | | |
| --- | --- | --- | --- | --- | --- |
|  | **Wild allele** | **Function** | **Variant allele** | **Function** | **Reference** |
| *GSTM1* | *Present* | ND | *Null* | Absent | [47] |
| *GSTT1* | *Present* | ND | *Null* | Absent | [48] |
| *GSTP1* c.313A>G | A | ND | G | RD | [49] |
| *XPC* c.2815A>C | A | NR | C | RR | [50] |
| *XPD* c.934G>A | G | NR | A | RR | [35] |
| *XPD* c.2251A>C | A | NR | C | RR | [35] |
| *XPF* c.2505T>C | T | NR | C | RR | [51] |
| *ERCC1* c.354C>T | C | NR | T | RR | [52] |
| *MLH1* c.93G>A | G | NR | A | RR | [53] |
| *MSH2* c.211+9C>G | C | NR | G | RR | [54] |
| *MSH3* c.3133A>G | A | NR | G | RR | [55] |
| *EXO1* c.1762G>A | G | NR | A | RR | [56] |
| *TP53* c.215G>C | G | NA | C | RA | [57] |
| *CASP3* c.-1191A>G | A | NA | G | RA | [42] |
| *CASP3* c.-182-247G>T | G | NA | T | RA | [43] |
| *FAS* c.-1378G>A | G | NA | A | RA | [58] |
| *FAS* c.-671A>G | A | NA | G | RA | [59] |
| *FASL* c.-844C>T | C | NA | T | RA | [60] |

SNV: single nucleotide variant; ND: normal detoxification; RD: reduced detoxification; NR: normal repair; RR: reduced repair; NA: normal apoptosis; RA: reduced apoptosis
