# Supplementary material for: Genetic Variability in Cisplatin Metabolism in Kidney Injury in Patients With Head and Neck Squamous Cell Carcinoma Undergoing Definitive Chemoradiotherapy
Source: Head Neck. 2025 May 8;47(10):2683–92. doi: 10.1002/hed.28179 (PMC12434574; doi:10.1002/hed.28179)
Supplement: Supplementary file 3 — Table S3. [file HED-47-2683-s002.doc]

**Supplementary Table S3**. Analysis of detoxification genotypes, DNA repair and apoptosis-related single nucleotide variants regarding nephrotoxicity in patients with head and neck squamous cell carcinoma after chemoradiotherapy.

| **Variable** | **N*** | **∆eGFR (%)** | ***P*-value** |
| --- | --- | --- | --- |
|  |  | **(Mean ± SD)** |  |
| **Age** |  |  |  |
| ≤ 56 years | 51 | 7.11 ± 19.63 | **0.08** |
| > 56 years | 58 | 0.42 ± 19.60 |  |
| **Gender** |  |  |  |
| Female | 8 | -0.23 ± 21.75 | 0.62 |
| Male | 101 | 3.85 ±19.74 |  |
| **Tobacco consumption** |  |  |  |
| Smokers | 106 | 3.46 ± 19.59 | 0.86 |
| Non-smokers | 3 | 7.06 ± 32.18 |  |
| **Alcohol consumption** |  |  |  |
| Drinkers | 100 | 3.16 ± 19.90 | 0.49 |
| Abstainers | 9 | 7.97 ± 19.40 |  |
| **Hypertension** |  |  |  |
| Yes | 28 | -0.22 ± 22.82 | 0.29 |
| No | 81 | 4.86 ± 18.64 |  |
| **Diabetes** |  |  |  |
| Yes | 12 | 0.98 ± 23.18 | 0.68 |
| No | 97 | 3.87 ± 19.47 |  |
| **BMI** |  |  |  |
| ≤ 19.6 | 63 | 3.16 ± 17.47 | 0.77 |
| > 19.6 | 46 | 4.10 ± 22.83 |  |
| **Tumor location** |  |  |  |
| Oral cavity or oropharynx | 28 | 5.15 ± 23.95 | 0.67 |
| Hypopharynx or larynx | 81 | 3.00 ± 18.30 |  |
| **Histological grade*** |  |  |  |
| Well or moderately differentiated | 73 | 3.64 ± 20.22 | 0.55 |
| Poorly or undifferentiated | 16 | 0.39 ± 24.25 |  |
| **Stage T** |  |  |  |
| T1 or T2 | 27 | 2.51 ± 18.37 | 0.74 |
| T3 or T4 | 82 | 3.90 ± 20.36 |  |
| **Stage N** |  |  |  |
| 0 or 1 | 42 | 3.72 ± 16.59 | 0.94 |
| 2 or 3 | 67 | 3.44 ± 21.71 |  |
| **Stage M** |  |  |  |
| M0 |  | NE |  |
| M1 |  |  |  |
| **Tumor stage** |  |  |  |
| I or II | 6 | 5.95 ± 4.73 | 0.37 |
| III or IV | 103 | 3.42 ± 20.35 |  |
| ***Isolated SNVs in detoxification genes*** | | | |
| ***GSTM1*** |  |  |  |
| Present | 48 | 2.13 ± 22.98 | 0.52 |
| Null | 61 | 4.68 ± 17.03 |  |
| ***GSTT1*** |  |  |  |
| Present | 92 | 5.39 ± 18.43 | **0.07** |
| Null | 17 | -6.39 ± 24.37 |  |
| ***GSTP1* c.313A>G** |  |  |  |
| AA | 54 | -0.05 ±21.43 | **0.06** |
| AG or GG | 55 | 7.09 ± 17.57 |  |
| AA or AG | 103 | 3.49 ± 19.86 | 0.90 |
| GG | 6 | 4.69 ±20.84 |  |
| ***Isolated SNVs in NER/MMR repair genes*** | | | |
| ***XPC* c.2815A>C** |  |  |  |
| AA | 41 | 0.92 ± 19.81 | 0.28 |
| AC or CC | 68 | 5.14 ± 19.79 |  |
| AA or AC | 96 | 3.63 ± 19.93 | 0.91 |
| CC | 13 | 2.99 ± 19.70 |  |
| ***XPD* c.934G>A** |  |  |  |
| GG | 59 | 4.90 ± 15.68 | 0.46 |
| GA or AA | 50 | 1.96 ±23.87 |  |
| GG or GA | 98 | 4.79 ± 19.13 | 0.12 |
| AA | 11 | -7.45 ± 23.27 |  |
| ***XPD* c.2251A>C** |  |  |  |
| AA | 55 | 5.42 ± 13.92 | 0.33 |
| AC or CC | 54 | 1.65 ±24.40 |  |
| AA or AC | 100 | 4.20 ± 19.67 | 0.32 |
| CC | 9 | - 3.56 ± 21.20 |  |
| ***XPF* c.2505T>C** |  |  |  |
| TT | 52 | 4.44 ± 18.91 | 0.66 |
| TC or CC | 57 | 2.75 ±20.74 |  |
| TT or TC | 101 | 3.61 ± 18.31 | 0.96 |
| CC | 8 | 2.89 ± 35.57 |  |
| ***ERCC1* c.354C>T** |  |  |  |
| CC | 28 | -3.09 ± 25.03 | **0.09** |
| CT or TT | 81 | 5.85 ± 17.26 |  |
| CC or CT | 89 | 2.66 ± 20.60 | 0.25 |
| TT | 20 | 7.52 ± 15.71 |  |
| ***MLH1* c.93G>A** |  |  |  |
| GG | 63 | 2.13 ± 19.60 | 0.39 |
| GA or AA | 46 | 5.50 ±20.15 |  |
| GG or GA | 105 | 2.73 ± 19.37 | 0.13 |
| AA | 4 | 25.23 ± 21.86 |  |
| ***MSH2* c.211+9C>G** |  |  |  |
| CC | 25 | - 0.71 ± 24.26 | 0.30 |
| CG or GG | 84 | 4.82 ±18.26 |  |
| CC or CG | 82 | 4.25 ±20.31 | 0.51 |
| GG | 27 | 1.45 ±18.42 |  |
| ***MSH3* c.3133A>G** |  |  |  |
| AA | 61 | 2.03 ± 20.54 | 0.36 |
| AG or GG | 48 | 5.49 ± 18.88 |  |
| AA or AG | 99 | 2.94 ± 19.97 | 0.29 |
| GG | 10 | 9.64 ± 17.95 |  |
| ***EXO1* c.1765G>A** |  |  |  |
| GG | 45 | 6.88 ± 20.87 | 0.15 |
| GA or AA | 64 | 1.22 ± 18.86 |  |
| GG or GA | 98 | 3.63 ± 20.06 | 0.90 |
| AA | 11 | 2.91 ±18.30 |  |
| ***Isolated SNVs in intrinsic/extrinsic apoptosis genes*** | | | |
| ***TP53* c.215G>C** |  |  |  |
| GG | 51 | 3.93 ± 18.09 | 0.85 |
| GC or CC | 58 | 3.23 ±21.37 |  |
| GG or GC | 98 | 2.63 ± 19.96 | 0.12 |
| CC | 11 | 11.82 ± 17.06 |  |
| ***FAS* c.-671A>G** |  |  |  |
| AA | 34 | 2.62 ± 20.08 | 0.74 |
| AG or GG | 75 | 3.98 ±19.81 |  |
| AA or AG | 83 | 3.66 ± 19.07 | 0.93 |
| GG | 26 | 3.22 ± 22.41 |  |
| ***FAS* c.-1378G>A** |  |  |  |
| GG | 80 | 4.22 ± 18.43 | 0.61 |
| GA or AA | 29 | 1.73 ± 23.48 |  |
| GG or GA | 102 | 3.89 ± 18.64 | 0.71 |
| AA | 7 | - 1.30 ± 34.44 |  |
| ***FASL* c.-844C>T** |  |  |  |
| CC | 32 | 2.75 ± 21.90 | 0.80 |
| CT or TT | 77 | 3.89 ± 19.03 |  |
| CC or CT | 82 | 4.73 ± 19.15 | 0.32 |
| TT | 27 | - 0.02 ± 21.70 |  |
| ***CASP3* c.-1191A>G** |  |  |  |
| AA | 45 | 3.12 ± 21.43 | 0.85 |
| GA or GG | 64 | 3.86 ± 18.76 |  |
| AA or GA | 98 | 3.50 ± 20.49 | 0.91 |
| GG | 11 | 4.01 ± 13.00 |  |
| ***CASP3* c.-182-247G>T** |  |  |  |
| GG | 39 | 1.12 ± 22.40 | 0.37 |
| GT or TT | 70 | 4.91 ± 18.25 |  |
| GG or GT | 96 | 3.84 ± 20.75 | 0.52 |
| TT | 13 | 1.42 ± 10.95 |  |
| ***Combined SNVs in detoxification genes*** | | | |
| ***GSTM1 + GSTT1*** |  |  |  |
| Present + Present | 41 | 4.36 ± 19.85 | 0.16 |
| Null + Null | 10 | -3.19 ± 13.31 |  |
| ***GSTM1* + *GSTP1* c.313A>G** |  |  |  |
| Present + AA | 21 | -7.73 ± 26.80 | **0.08** |
| Null + AG or GG | 28 | 4.48 ± 18.71 |  |
| Present + AA or AG | 44 | 2.11 ± 23.08 | 0.58 |
| Null + GG | 2 | 9.46 ± 13.37 |  |
| ***GSTT1 + GSTP1* c.313A>G** |  |  |  |
| Present + AA | 45 | 2.81 ± 19.61 | 0.97 |
| Null + AG or GG | 8 | 2.54 ± 20.96 |  |
| Present + AA or AG |  | NE |  |
| Null + GG |  |  |  |
| ***Combined SNVs in detoxification and NER/MMR repair genes*** | | | |
| ***GSTM1 + XPC* c.2815A>C** |  |  |  |
| Present + AA | 20 | 0.29 ± 25.67 | 0.36 |
| Null + AC or CC | 40 | 6.33 ± 18.89 |  |
| Present + AA or AC | 41 | 2.34 ± 22.66 | 0.52 |
| Null + CC | 4 | 5.48 ± 7.86 |  |
| ***GSTM1 + XPD* c.934G>A** |  |  |  |
| Present + GG | 25 | 7.10 ± 17.92 | 0.90 |
| Null + GA or AA | 27 | 6.43 ± 20.49 |  |
| Present + GG or GA | 43 | 4.66 ± 21.33 | 0.77 |
| Null + AA | 6 | 2.71 ± 13.97 |  |
| ***GSTM1 + XPD* c.2251A>C** |  |  |  |
| Present + AA | 24 | 8.81 ± 15.20 | 0.67 |
| Null + AC or CC | 30 | 6.62 ± 20.78 |  |
| Present + AA or AC | 43 | 3.60 ± 22.44 | 0.79 |
| Null + CC | 4 | 5.21 ± 9.33 |  |
| ***GSTM1 + XPF* c.2505T>C** |  |  |  |
| Present + TT | 31 | 4.58 ± 19.57 | 0.92 |
| Null + TC or CC | 22 | 3.96 ± 24.23 |  |
| Present + TT or TC | 44 | 3.68 ± 22.19 | 0.42 |
| Null + CC | 4 | 20.76 ± 36.18 |  |
| ***GSTM1 + ERCC1* c.354C>T** |  |  |  |
| Present + CC | 14 | -7.15 ± 27.22 | 0.11 |
| Null + CT or TT | 47 | 5.79 ± 14.98 |  |
| Present + CC or CT | 40 | 0.60 ± 24.28 | 0.40 |
| Null + TT | 12 | 6.03 ± 17.43 |  |
| ***GSTM1 + MLH1* c.93G>A** |  |  |  |
| Present + GG | 31 | 0.04 ± 20.24 | 0.26 |
| Null + GA or AA | 29 | 5.26 ± 14.77 |  |
| Present + GG or GA | 47 | 1.59 ± 22.93 | 0.27 |
| Null + AA | 3 | 24.52 ± 26.71 |  |
| ***GSTM1 + MSH2* c.211+9C>G** |  |  |  |
| Present + CC | 13 | 3.02 ± 30.31 | 0.66 |
| Null + CG or GG | 49 | 6.99 ± 16.67 |  |
| Present + CC or CG | 37 | 1.57 ± 21.95 | 0.66 |
| Null + GG | 16 | -0.29 ± 9.20 |  |
| ***GSTM1 + MSH3* c.3133A>G** |  |  |  |
| Present + AA | 28 | 2.26 ± 25.02 | 0.32 |
| Null + AG or GG | 28 | 8.03 ± 17.63 |  |
| Present + AA or AG | 44 | 1.86 ± 23.67 | 0.27 |
| Null + GG | 6 | 12.70 ± 20.26 |  |
| ***GSTM1 + EXO1* c.1765G>A** |  |  |  |
| Present + GG | 17 | 4.49 ± 26.79 | 0.69 |
| Null + GA or AA | 33 | 1.58 ± 16.95 |  |
| Present + GG or GA | 42 | 1.41 ± 23.71 | 0.72 |
| Null + AA | 5 | -2.18 ± 19.34 |  |
| ***GSTT1 + XPC* c.2815A>C** |  |  |  |
| Present + AA | 34 | 3.17 ± 17.58 | 0.39 |
| Null + AC or CC | 10 | -3.87 ± 23.22 |  |
| Present + AC or AA | 81 | 5.06 ± 19.07 | 0.45 |
| Null + CC | 2 | -23.62 ± 35.26 |  |
| ***GSTTI + XPD* c.934G>A** |  |  |  |
| Present + GG | 51 | 5.39 ± 15.27 | 0.07 |
| Null + GA or AA | 9 | -13.70 ± 27.34 |  |
| Present + GG or GA |  | NE |  |
| Null + AA |  |  |  |
| ***GSTT1 + XPD* c.2251A>C** |  |  |  |
| Present + AA | 47 | 5.60 ± 12.89 | 0.03 |
| Null + AC or CC | 9 | -15.96 ± 24.87 |  |
| Present + AA or AC | 85 | 6.25 ± 17.78 | 0.09 |
| Null + CC | 2 | 1.69 ± 1.97 |  |
| ***GSTT1 + XPF* c.2505T>C** |  |  |  |
| Present + TT | 45 | 5.87 ± 17.25 | 0.12 |
| Null + TC or CC | 10 | -7.53 ± 23.58 |  |
| Present + TT or TC | 86 | 5.52 ± 16.18 | 0.73 |
| Null + CC | 2 | 0.83 ± 14.42 |  |
| ***GSTT1 + ERCC1* c.354C>T** |  |  |  |
| Present + CC | 24 | -0.48 ± 24.93 | 0.81 |
| Null + CT or TT | 13 | -2.57 ± 24.55 |  |
| Present + CC or CT | 74 | 4.20 ± 19.27 | 0.12 |
| Null + TT | 2 | -17.31 ± 8.02 |  |
| ***GSTT1 + MLH1* c.93G>A** |  |  |  |
| Present + GG | 54 | 3.94 ± 19.50 | 0.52 |
| Null + GA or AA | 8 | -3.75 ± 31.55 |  |
| Present + GG or GA |  | NE |  |
| Null + AA |  |  |  |
| ***GSTT1 + MSH2* c.211+9C>G** |  |  |  |
| Present + CC | 20 | -0.46 ± 20.69 | 0.26 |
| Null + CG or GG | 12 | -8.34 ± 17.49 |  |
| Present + CC or CG | 70 | 5.32 ± 19.32 | 0.07 |
| Null + GG | 5 | -16.90 ± 20.12 |  |
| ***GSTT1 + MSH3* c.3133A>G** |  |  |  |
| Present + AA | 54 | 3.47 ± 19.23 | 0.32 |
| Null + AG or GG | 10 | -4.52 ± 22.80 |  |
| Present + AA or AG | 84 | 4.72 ± 18.36 | 0.54 |
| Null + GG | 2 | -1.52 ± 10.27 |  |
| ***GSTT1 + EXO1* c.1762G>A** |  |  |  |
| Present + GG | 37 | 9.16 ± 18.26 | 0.03 |
| Null + GA or AA | 9 | -8.80 ± 20.40 |  |
| Present + GG or GA |  | NE |  |
| Null + AA |  |  |  |
| ***GSTP1* c.313A>G *+ XPC* c.2815A>C** |  |  |  |
| AA + AA | 21 | -2.04 ± 22.52 | **0.07** |
| AG or GG + AC or CC | 35 | 8.83 ± 18.15 |  |
| AA or AG + AA or AC |  | NE |  |
| CC + CC |  |  |  |
| ***GSTP1* c.313A>G *+ XPD* c.934G>A** |  |  |  |
| AA + GG | 31 | 3.27 ± 17.06 | 0.41 |
| AG or GG + GA or AA | 27 | 7.48 ± 20.87 |  |
| AA or AG + GG or GA | 94 | 4.59 ± 19.30 | 0.76 |
| GG + AA | 2 | -4.80 ± 33.53 |  |
| ***GSTP1* c.313A>G *+ XPD* c.2251A>C** |  |  |  |
| AA + AA | 28 | 3.46 ± 12.43 | 0.46 |
| AG or GG + AC or CC | 28 | 6.72 ± 19.81 |  |
| AA or AG + AA or AC | 96 | 3.98 ± 19.85 | 0.77 |
| AA + CC | 2 | -4.80 ± 33.53 |  |
| ***GSTP1* c.313A>G *+ XPF* c.2505T> C** |  |  |  |
| AA + TT | 26 | -1.38 ± 19.16 | 0.27 |
| AG or GG + TC or CC | 29 | 4.26 ± 17.80 |  |
| AA or AG + TT or TC |  | NE |  |
| GG or CC |  |  |  |
| ***GSTP1* c.313A>G *+ ERCC1* c.354C>T** |  |  |  |
| AA + CC | 13 | -7.78 ± 28.08 | **0.05** |
| AG or GG + CT or TT | 40 | 9.38 ± 15.18 |  |
| AA or AG + CC or CT |  | NE |  |
| GG + TT |  |  |  |
| ***GSTP1* c.313A>G *+ MLH1* c.93G>A** |  |  |  |
| AA + GG | 30 | 1.26 ± 21.81 | **0.02** |
| AG or GG + GA or AA | 22 | 13.33 ± 15.83 |  |
| AA or AG + GG or GA |  | NE |  |
| GG + AA |  |  |  |
| ***GSTP1* c.313A>G *+ MSH2* c.211+9C>G** |  |  |  |
| AA + CC | 14 | -5.96 ± 23.29 | **0.06** |
| AG or GG + CG or GG | 44 | 7.37 ± 15.60 |  |
| AA or AG + CC or CG | 78 | 4.13 ± 20.15 | 0.22 |
| GG + GG | 2 | 1.02 ± 1.44 |  |
| ***GSTP1* c.313A>G *+ MSH3* c.3133A>G** |  |  |  |
| AA + AA | 27 | -2.81 ± 22.05 | **0.03** |
| AG or GG + AG or GG | 21 | 9.07 ± 15.80 |  |
| AA or AG + AA or AG |  | NE |  |
| GG + GG |  |  |  |
| ***GSTP1* c.313A>G *+ EXO1* c.1762G>A** |  |  |  |
| AA + GG | 22 | 1.23 ± 24.76 | 0.73 |
| AG or GG + GA or AA | 32 | 3.36 ± 18.58 |  |
| AA or AG + GG or GA |  | NE |  |
| GG or AA |  |  |  |
| ***Combined SNVs in detoxification and intrinsic/extrinsic apoptosis genes*** | | | |
| ***GSTM1 + TP53* c.215G>C** |  |  |  |
| Present + CC | 5 | 15.71 ± 21.05 | 0.30 |
| Null + GC or GG | 55 | 4.25 ± 17.37 |  |
| Present + CC or GC | 27 | 0.89 ± 24.43 | 0.57 |
| Null + GG | 30 | 4.08 ± 15.70 |  |
| ***GSTM1 + FAS* c.-671A>G** |  |  |  |
| Present + GG | 10 | 0.92 ± 29.54 | 0.70 |
| Null + AG or AA | 45 | 4.69 ± 17.04 |  |
| Present + GG or AG | 27 | 3.66 ± 22.84 | 0.60 |
| Null + AA | 13 | 6.60 ± 12.44 |  |
| ***GSTM1 + FAS* c.-1378G>A** |  |  |  |
| Present + AA | 5 | -9.10 ± 34.16 | 0.43 |
| Null + GA or GG | 59 | 4.22 ± 16.42 |  |
| Present + AA or GA | 10 | -10.20 ± 25.70 | 0.14 |
| Null + GG | 42 | 3.17 ± 15.44 |  |
| ***GSTM1 + FASL* c.-844C>T** |  |  |  |
| Present + TT | 13 | 1.66 ± 29.13 | 0.57 |
| Null + CT or TT | 47 | 6.54 ± 17.87 |  |
| Present + TT or CT | 36 | 1.68 ± 22.37 | 0.92 |
| Null + CC | 20 | 2.31 ± 19.95 |  |
| ***GSTM1 + CASP3* c.-1191A>G** |  |  |  |
| Present + GG | 6 | 6.44 ± 7.19 | 0.70 |
| Null + GA or AA | 56 | 5.00 ± 17.04 |  |
| Present + GG or GA | 25 | 2.41 ± 22.37 | 0.73 |
| Null + AA | 22 | 4.48 ± 18.67 |  |
| ***GSTM1 + CASP3* c.-182-247G>T** |  |  |  |
| Present + TT | 2 | 2.60 ± 4.80 | 0.56 |
| Null + TT or GT | 50 | 5.44 ± 17.97 |  |
| Present + TT or GT | 29 | 5.94 ± 19.50 | 0.96 |
| Null + GG | 20 | 5.69 ± 16.36 |  |
| ***GSTT1 + TP53* c.215G>C** |  |  |  |
| Present + CC | 10 | 8.86 ± 14.70 | **0.02** |
| Null + GC or GG | 16 | -9.38 ± 21.71 |  |
| Present + CC or GC | 8 | -6.54 ± 11.45 | 0.03 |
| Null + GG | 49 | 4.97 ± 18.53 |  |
| ***GSTT1 + FAS* c.-671A>G** |  |  |  |
| Present + GG | 23 | 7.75 ± 17.55 | 0.20 |
| Null + AG or AA | 14 | -1.01 ± 20.60 |  |
| Present + GG or AG | 65 | 5.14 ± 18.26 | 0.08 |
| Null + AA | 7 | -10.41 ± 19.40 |  |
| ***GSTT1 + FAS* c.-1378G>A** |  |  |  |
| Present + AA | 5 | 12.82 ± 22.82 | 0.23 |
| Null + GA or GG | 15 | -2.37 ± 20.51 |  |
| Present + AA or GA | 24 | 6.11 ± 20.81 | 0.37 |
| Null + GG | 12 | -1.02 ± 22.40 |  |
| ***GSTT1 + FASL* c.-844C>T** |  |  |  |
| Present + TT | 24 | 2.68 ± 18.26 | 0.40 |
| Null + CT or CC | 14 | -3.12 ± 20.86 |  |
| Present + TT or CT | 65 | 5.99 ± 18.23 | 0.58 |
| Null + CC | 5 | -3.75 ± 35.52 |  |
| ***GSTT1 + CASP3* c.-1191A>G** |  |  |  |
| Present + GG | 10 | 3.30 ± 13.48 | 0.16 |
| Null + AG or AA | 16 | -7.48 ± 24.74 |  |
| Present + GG or AG | 54 | 6.68 ± 15.68 | 0.48 |
| Null + AA | 7 | 0.71 ± 20.42 |  |
| ***GSTT1 + CASP3* c.-182-247G>T** |  |  |  |
| Present + TT | 10 | 4.73 ± 7.77 | 0.18 |
| Null + GT or GG | 14 | -5.71 ± 26.38 |  |
| Present + TT or GT | 58 | 5.71 ± 18.56 | 0.10 |
| Null + GG | 5 | -24.29 ± 32.07 |  |
| ***GSTP1* c.313A>G *+ TP53* c.215G>C** |  |  |  |
| AA + CC | 4 | 3.95 ± 13.27 | 0.81 |
| AG or GG + GC or CC | 48 | 5.74 ± 17.26 |  |
| AA or AG + CC or GC | 56 | 3.85 ± 21.31 | 0.28 |
| GG + GG | 4 | 14.16 ± 15.14 |  |
| ***GSTP1* c.313A>G *+ FAS* c.-671A>G** |  |  |  |
| AA + GG | 12 | 1.45 ± 27.16 | 0.45 |
| AG or GG + AG or AA | 41 | 7.90 ± 17.46 |  |
| AA or AG + GG or AG | 71 | 4.31 ± 19.91 | 0.55 |
| GG + AA | 2 | 17.78 ± 22.26 |  |
| ***GSTP1* c.313A>G *+ FAS* c.-1378G>A** |  |  |  |
| AA + AA | 3 | -24.81 ± 34.50 | 0.26 |
| AG or GG + GA or GG | 51 | 6.36 ± 17.01 |  |
| AA or AG or AA or GA | 27 | 1.08 ± 24.12 | 0.96 |
| GG + GG | 4 | 1.76 ± 25.35 |  |
| ***GSTP1* c.313A>G *+ FASL* c.-844C>T** |  |  |  |
| AA + TT | 16 | -6.60 ± 23.60 | **0.06** |
| AG or GG + CT or CC | 44 | 6.47 ± 18.30 |  |
| AA or AG + TT or CT | 74 | 3.99 ± 19.40 | 0.85 |
| GG + CC | 3 | 7.97 ± 32.43 |  |
| ***GSTP1* c.313A>G *+ CASP3* c.-1191A>G** |  |  |  |
| AA + GG | 4 | 2.76 ± 11.76 | 0.51 |
| AG or GG + AG or AA | 48 | 7.43 ± 18.08 |  |
| AA or AG or GG or AG | 62 | 3.41 ± 18.68 | 0.64 |
| GG + AA | 4 | -1.85 ± 19.68 |  |
| ***GSTP1* c.313A>G *+ CASP3* c.-182-247G>T** |  |  |  |
| AA + TT | 8 | 2.92 ± 9.73 | 0.26 |
| AG or GG + GT or GG | 50 | 7.89 ± 17.84 |  |
| AA or AG + TT or GT | 66 | 5.33 ± 18.24 | 0.57 |
| GG + GG | 2 | 17.85 ± 22.16 |  |
| ***Combined SNVs in NER/MMR repair genes*** | | | |
| ***XPC* c.2815A>C *+ XPD* c.934G>A** |  |  |  |
| AA + GG | 25 | 3.82 ± 14.39 | 0.88 |
| AC or CC + GA or AA | 34 | 4.58 ± 22.69 |  |
| AA or AC + GG or GA | 87 | 5.14 ± 18.98 | 0.77 |
| GG + AA | 2 | 8.49 ± 12.73 |  |
| ***XPC* c.2815A>C *+ XPD* c.2251A>C** |  |  |  |
| AA + AA | 24 | 3.76 ± 14.68 | 0.99 |
| AC or CC + AC or CC | 37 | 3.83 ± 23.99 |  |
| AA or AC + AA or AC |  | NE |  |
| CC or CC |  |  |  |
| ***XPC* c.2815A>C *+ XPF* c.2505T> C** |  |  |  |
| AA + TT | 24 | 4.09 ± 19.43 | 0.75 |
| AC or CC + TC or CC | 40 | 5.43 ± 18.34 |  |
| AA or AC + TT or TC |  | NE |  |
| CC or CC |  |  |  |
| ***XPC* c.2815A>C *+ ERCC1* c.354C>T** |  |  |  |
| AA + CC | 8 | -6.34 ± 27.61 | 0.19 |
| AC or CC + CT or TT | 48 | 8.03 ± 16.90 |  |
| AA or AC + CC or CT |  | NE |  |
| CC or TT |  |  |  |
| ***XPC* c.2815A>C *+ MLH1* c.93G>A** |  |  |  |
| AA + GG | 26 | 0.44 ± 17.48 | 0.15 |
| AC or CC + GA or AA | 31 | 7.31 ± 18.17 |  |
| AA or AC + GG or GA |  | NE |  |
| CC or AA |  |  |  |
| ***XPC* c.2815A>C *+ MSH2* c.211+9C>G** |  |  |  |
| AA + CC | 10 | -2.77 ± 24.92 | 0.29 |
| AC or CC + GC or GG | 53 | 6.41 ± 18.29 |  |
| AA or AC + CC or GC | 74 | 3.82 ± 20.83 | 0.46 |
| CC + GG | 5 | -5.32 ± 24.95 |  |
| ***XPC* c.2815A>C *+ MSH3* c.3133A>G** |  |  |  |
| AA + AA | 21 | 0.22 ± 26.20 | 0.27 |
| AC or CC + AG or GG | 28 | 8.24 ± 23.01 |  |
| AA or AC + AA or AG |  | NE |  |
| CC+ GG |  |  |  |
| ***XPC* c.2815A>C *+ EXO1* c.1762G>A** |  |  |  |
| AA + GG | 12 | -2.58 ± 23.88 | 0.72 |
| AC or CC + GA or AA | 35 | 0.26 ± 19.63 |  |
| AA or AC + GG or GA |  | NE |  |
| CC + AA |  |  |  |
| ***XPD* c.934G>A *+ XPD* c.2251A>C** |  |  |  |
| GG + AA | 47 | 4.80 ± 14.19 | 0.35 |
| GA or AA + AC or CC | 42 | 0.60 ± 25.35 |  |
| GG or AG + AA or AC | 94 | 4.81 ± 19.51 | 0.30 |
| AA + CC | 5 | -9.78 ± 27.56 |  |
| ***XPD* c.934G>A *+ XPF* c.2505T>C** |  |  |  |
| GG + TT | 29 | 4.39 ± 16.59 | 0.44 |
| AG or AA + TC or CC | 27 | -0.19 ± 25.65 |  |
| GG or AG + TT or TC | 93 | 4.04 ± 18.20 | 0.22 |
| AA + CC | 3 | -23.64 ± 27.14 |  |
| ***XPD* c.934G>A *+ ERCC1* c.354C>T** |  |  |  |
| GG + CC | 13 | 1.86 ± 23.96 | 0.60 |
| AG or AA + CT or TT | 35 | 5.97 ± 22.11 |  |
| GG or AG + CC or CT |  | NE |  |
| AA + TT |  |  |  |
| ***XPD* c.934G>A *+ MLH1* c.93G>A** |  |  |  |
| GG + GG | 33 | 3.80 ± 14.86 | 0.91 |
| GA or AA + GA or AA | 20 | 4.46 ± 24.21 |  |
| GG or GA + GG or GA |  | NE |  |
| AA + AA |  |  |  |
| ***XPD* c.934G>A *+ MSH2* c.211+9C>G** |  |  |  |
| GG + CC | 16 | 4.66 ± 14.91 | 1.00 |
| GA or AA + CG or GG | 41 | 4.64 ± 20.47 |  |
| GG or AG + CC or CG | 73 | 5.96 ± 19.12 | 0.45 |
| AA + GG | 2 | 2.55 ± 4.33 |  |
| ***XPD* c.934G>A *+ MSH3* c.3133A>G** |  |  |  |
| GG + AA | 32 | 3.32 ± 15.47 | 0.93 |
| GA or AA + AG or GG | 21 | 3.84 ± 22.35 |  |
| GG or GA + AA or AG |  | NE |  |
| AA + GG |  |  |  |
| ***XPD* c.934G>A *+ EXO1* c.1765G>A** |  |  |  |
| GG + GG | 23 | 6.92 ± 20.08 | 0.17 |
| GA or AA + GA or AA | 28 | -1.87 ± 24.87 |  |
| GG or GA + GG or GA |  | NE |  |
| AA + AA |  |  |  |
| ***XPD* c.2251A>C *+ XPF* c.2505T>C** |  |  |  |
| AA + TT | 26 | 6.37 ± 15.40 | 0.36 |
| AC or CC + TC or CC | 28 | 0.86 ± 26.81 |  |
| AA or AC + TT or TC | 95 | 3.43 ± 18.79 | 0.22 |
| CC + CC | 3 | -23.64 ± 27.14 |  |
| ***XPD* c.2251A>C *+ ERCC1* c.354C>T** |  |  |  |
| AA + CC | 8 | -1.41 ± 16.48 | 0.38 |
| AC or CC + CT or TT | 34 | 4.83 ± 21.77 |  |
| AA or AC + CC or CT |  | NE |  |
| CC or TT |  |  |  |
| ***XPD* c.2251A>C *+ MLH1* c.93G>A** |  |  |  |
| AA + GG | 31 | 4.50 ± 12.52 | 0.97 |
| AC or CC + GA or AA | 22 | 4.29 ± 24.41 |  |
| AA or AC + GG or GA |  | NE |  |
| CC or AA |  |  |  |
| ***XPD* c.2251A>C *+ MSH2* c.211+9C>G** |  |  |  |
| AA + CC | 14 | 4.00 ± 15.51 | 0.97 |
| AC or CC + CG or GG | 43 | 3.79 ± 21.98 |  |
| AA or AC + CC or CG | 75 | 5.32 ± 19.89 | 0.46 |
| CC + GG | 2 | 9.13 ± 4.98 |  |
| ***XPD* c.2251A>C *+ MSH3* c.3133A>G** |  |  |  |
| AA + AA | 28 | 5.71 ± 14.21 | 0.97 |
| AC or CC + AG or GG | 21 | 5.96 ± 24.24 |  |
| AA or AC + AA or AG |  | NE |  |
| CC + GG |  |  |  |
| ***XPD* c.2251A>C *+ EXO1* c.1765G>A** |  |  |  |
| AA + GG | 20 | 8.02 ± 15.97 | **0.09** |
| AC or CC + GA or AA | 29 | -2.07 ± 24.22 |  |
| AA or AC + GG or GA |  | NE |  |
| CC + AA |  |  |  |
| ***XPF* c.2505T>C *+ ERCC1* c.354C>T** |  |  |  |
| TT + CC | 12 | -2.90 ± 25.26 | 0.32 |
| TC or CC + CT or TT | 41 | 5.09 ± 18.30 |  |
| TT or TC + CC or CT |  | NE |  |
| CC or TT |  |  |  |
| ***XPF* c.2505T>C *+ MLH1* c.93G>A** |  |  |  |
| TT + GG | 30 | 2.70 ± 18.75 | 0.77 |
| TC or CC + GA or AA | 24 | 4.31 ± 21.24 |  |
| TT or TC + GG or GA |  | NE |  |
| CC or AA |  |  |  |
| ***XPF* c.2505T>C *+ MSH2* c.211+9C>G** |  |  |  |
| TT + CC | 12 | 5.18 ± 20.63 | 0.98 |
| TC or CC + CG or GG | 44 | 5.38 ± 18.11 |  |
| TT or TC + CC or CG | 76 | 4.23 ± 18.16 | 0.56 |
| CC + GG | 2 | -1.88 ± 10.58 |  |
| ***XPF* c.2505T>C *+ MSH3* c.3133A>G** |  |  |  |
| TT + AA | 18 | 6.68 ± 18.58 | 0.57 |
| TC or CC + AG or GG | 22 | 10.00 ± 17.78 |  |
| TT or TC + AA or AG |  | NE |  |
| CC + GG |  |  |  |
| ***XPF* c.2505T>C *+ EXO1* c.1765G>A** |  |  |  |
| TT + GG | 22 | 3.75 ± 17.06 | 0.22 |
| TC or CC + GA or AA | 34 | -2.07 ± 16.97 |  |
| TT or TC + GG or GA |  | NE |  |
| CC + AA |  |  |  |
| ***ERCC1* c.354C>T *+ MLH1* c.93G>A** |  |  |  |
| CC + GG | 15 | -13.09 ± 24.13 | **0.02** |
| CT or TT + GA or AA | 33 | 4.34 ± 19.83 |  |
| CC or CT + GG or GA |  | NE |  |
| TT + AA |  |  |  |
| ***ERCC1* c.354C>T *+ MSH2* c.211+9C>G** |  |  |  |
| CC + CC | 7 | -7.03 ± 22.02 | 0.15 |
| CT or TT + CG or GG | 63 | 7.03 ± 14.26 |  |
| CC or CT+ CC or CG | 67 | 3.13 ± 20.97 | 0.90 |
| TT + GG | 5 | 2.35 ± 11.85 |  |
| ***ERCC1* c.354C>T *+ MSH3* c.3133A>G** |  |  |  |
| CC + AA | 16 | -6.07 ± 25.42 | **0.07** |
| CT or TT + AG or GG | 36 | 7.03 ± 16.50 |  |
| CC or CT+ AA or AG | 81 | 1.75 ± 20.58 | 0.62 |
| TT + GG | 2 | 0.58 ± 0.82 |  |
| ***ERCC1* c.354C>T *+ EXO1* c.1765G>A** |  |  |  |
| CC + GG | 12 | 6.24 ± 23.02 | 0.86 |
| CT or TT+ GA or AA | 48 | 4.99 ± 14.89 |  |
| CC or CT + GG or GA | 81 | 2.35 ± 20.82 | 0.52 |
| TT + AA | 3 | -4.95 ± 16.13 |  |
| ***MLH1* c.93G>A *+ MSH2* c.211+9C>G** |  |  |  |
| GG + CC | 13 | 1.57 ± 20.38 | 0.28 |
| GA or AA + GC or GG | 34 | 8.57 ± 15.62 |  |
| GG or GA + CC or CG |  | NE |  |
| AA + GG |  |  |  |
| ***MLH1* c.93G>A *+ MSH3* c.3133A>G** |  |  |  |
| GG + AA | 37 | 0.37 ± 20.98 | 0.28 |
| GA or AA + AG or GG | 22 | 6.51 ± 20.72 |  |
| GG or GA + AA or AG |  | NE |  |
| AA + GG |  |  |  |
| ***MLH1* c.93G>A *+ EXO1* c.1762G>A** |  |  |  |
| GG + GG | 22 | 4.59 ± 18.73 | 0.62 |
| AG or AA + GA or AA | 23 | 1.94 ± 16.68 |  |
| GG or GA + GG or GA |  | NE |  |
| AA + AA |  |  |  |
| ***MSH2* c.211+9C>G *+ MSH3* c.3133A>G** |  |  |  |
| CC + AA | 17 | -2.65 ± 25.31 | 0.16 |
| CG or GG + AG or GG | 40 | 5.91 ± 18.28 |  |
| CC or GC + AA or AG | 74 | 3.80 ± 20.52 | 0.55 |
| GG + GG | 2 | 14.51 ± 17.64 |  |
| ***MSH2* c.211+9C>G *+ EXO1* c.1765G>A** |  |  |  |
| CC + GG | 10 | 5.21 ± 29.45 | 0.83 |
| GC or GG + GA or AA | 49 | 3.02 ± 18.25 |  |
| CC or GC + GG or GA | 74 | 4.85 ± 20.74 | 0.56 |
| GG + AA | 3 | 14.12 ± 23.27 |  |
| ***MSH3* c.3133A>G *+ EXO1* c.1762G>A** |  |  |  |
| AA + GG | 25 | 2.38 ± 21.97 | 0.73 |
| AG or GG + GA or AA | 28 | 0.48 ± 17.89 |  |
| AA or AG + GG or GA |  | NE |  |
| GG + AA |  |  |  |
| ***Combined SNVs in NER/MMR repair and apoptosis genes*** | | | |
| ***XPC* c.2815A>C *+ TP53* c.215G>C** |  |  |  |
| AA + CC | 5 | 4.93 ± 13.98 | 0.89 |
| AC or CC + GC or CC | 62 | 3.94 ± 19.64 |  |
| AA or AC + GC or CC | 50 | 3.47 ± 21.49 | 0.86 |
| CC + GG | 5 | 5.05 ± 17.67 |  |
| ***XPC* c.2815A>C *+ FAS* c.-671A>G** |  |  |  |
| AA + GG | 12 | -5.11 ± 27.07 | 0.30 |
| AC or CC+ AG or AA | 54 | 3.79 ± 20.75 |  |
| AA or AC+ GG or AG | 67 | 3.92 ± 20.68 | 0.82 |
| CC + AA | 5 | 0.59 ± 30.77 |  |
| ***XPC* c.2815A>C *+ FAS* c.-1378G>A** |  |  |  |
| AA + AA | 3 | -24.59 ± 34.66 | 0.28 |
| AC or CC+ GA or GG | 64 | 4.45 ± 19.46 |  |
| AA or AC+ GA or AA | 25 | 0.59 ± 25.03 | 0.98 |
| CC + GG | 9 | 0.38 ± 23.16 |  |
| ***XPC* c.2815A>C *+ FASL* c.-844C>T** |  |  |  |
| AA + TT | 14 | -7.89 ± 24.59 | 0.10 |
| AC or CC + CT or CC | 55 | 4.36 ± 20.85 |  |
| AA or AC + CT or TT | 70 | 3.87 ± 19.69 | 0.86 |
| CC + CC | 6 | 1.74 ± 27.89 |  |
| ***XPC* c.2815A>C *+ CASP3* c.-1191A>G** |  |  |  |
| AA + GG | 2 | -0.53 ± 1.81 | 0.07 |
| AC or CC + GG or AG | 59 | 5.16 ± 20.60 |  |
| AA or AC + GG or AG | 56 | 4.10 ± 18.03 | 0.98 |
| CC + AA | 5 | 4.29 ± 9.63 |  |
| ***XPC* c.2815A>C *+ CASP3* c.-182-247G>T** |  |  |  |
| AA + TT | 3 | 2.40 ± 12.66 | 0.69 |
| AC or CC + GT or GG | 58 | 5.83 ± 20.92 |  |
| AA or AC + TT or GT | 64 | 5.07 ± 18.78 | 0.83 |
| CC + GG | 7 | 2.81 ± 25.55 |  |
| ***XPD* c.934G>A *+ TP53* c.215G>C** |  |  |  |
| GG + CC | 6 | 14.63 ± 17.54 | 0.13 |
| GA or AA + GC or GG | 45 | 1.24 ± 24.50 |  |
| GG or GA + CC or GC | 51 | 4.82 ± 20.73 | 0.46 |
| AA + GG | 4 | -5.89 ± 25.31 |  |
| ***XPD* c.934G>A *+ FAS* c.-671A>G** |  |  |  |
| GG + AA | 13 | 4.19 ± 14.31 | 0.67 |
| GA or AA + AG or GG | 37 | 1.86 ± 22.26 |  |
| GG or GA + AA or AG | 69 | 4.72 ± 19.98 | 0.33 |
| AA + GG | 5 | -10.96 ± 31.13 |  |
| ***XPD* c.934G>A *+ FAS* c.-1378G>A** |  |  |  |
| GG + GG |  | NE |  |
| GA or AA + GA or AA |  |  |  |
| GG or GA + GG or GA | 25 | 1.30 ± 24.57 | 0.17 |
| AA +AA | 7 | -14.27 ± 24.53 |  |
| ***XPD* c.934G>A *+ FASL* c.-844C>T** |  |  |  |
| GG + TT | 15 | 2.92 ± 14.66 | 0.87 |
| GA or AA + CT or CC | 38 | 3.75 ± 22.34 |  |
| GG or AG + TT or CT | 71 | 5.42 ± 17.95 | 0.66 |
| AA + CC | 5 | 0.69 ± 22.25 |  |
| ***XPD* c.934G>A *+ CASP3* c.-1191A>G** |  |  |  |
| GG + GG | 6 | -2.34 ± 9.19 | 0.55 |
| GA or AA + AG or AA | 45 | 0.89 ± 24.61 |  |
| GG or GA + GG or AG | 58 | 3.97 ± 19.35 | 0.13 |
| AA + AA | 5 | -19.80 ± 28.23 |  |
| ***XPD* c.934G>A *+ CASP3* c.-182-247G>T** |  |  |  |
| GG + TT | 9 | 3.58 ± 7.67 | 0.80 |
| AG or AA + GT or GG | 46 | 2.43 ± 24.48 |  |
| GG or AG + TT or GT | 64 | 5.84 ± 18.24 | 0.31 |
| AA + GG | 5 | -10.46 ± 31.43 |  |
| ***XPD* c.2251A>C *+ TP53* c.215G>C** |  |  |  |
| AA + CC | 7 | 13.62 ± 19.28 | 0.16 |
| AC or CC + GC or GG | 50 | 1.09 ± 25.04 |  |
| AA or AC + CC or GC | 52 | 4.74 ± 20.84 | 0.49 |
| CC + GG | 3 | 9.12 ± 8.56 |  |
| ***XPD* c.2251A>C *+ FAS* c.-671A>G** |  |  |  |
| AA + GG | 11 | 2.07 ± 14.81 | 0.82 |
| AC or CC + AG or AA | 39 | 0.72 ± 23.56 |  |
| AA or AC + GG or AG | 69 | 4.22 ± 20.16 | 0.42 |
| CC + AA | 3 | -13.19 ± 30.19 |  |
| ***XPD* c.2251A>C *+ FAS* c.-1378G>A** |  |  |  |
| AA + AA |  | NE |  |
| AC or CC + GA or GG |  |  |  |
| AA or AC + AA or GA | 27 | 1.15 ± 24.12 | 0.40 |
| CC + GG | 7 | -7.33 ± 22.27 |  |
| ***XPD* c.2251A>C *+ FASL* c.-844C>T** |  |  |  |
| AA + TT | 17 | 4.77 ± 15.50 | 0.86 |
| AC or CC + CT or CC | 44 | 3.88 ± 23.14 |  |
| AA or AC + TT or CT | 70 | 4.60 ± 18.90 | 0.76 |
| CC + CC | 2 | -4.80 ± 33.53 |  |
| ***XPD* c.2251A>C *+ CASP3* c.-1191A>G** |  |  |  |
| AA + GG | 6 | 2.27 ± 17.12 | 0.89 |
| AC or CC + AG or AA | 49 | 1.20 ± 25.52 |  |
| AA or AC + GG or AG | 59 | 3.76 ± 19.51 | 0.31 |
| CC + AA | 4 | -14.33 ± 29. 75 |  |
| ***XPD* c.2251A>C *+ CASP3* c.-182-247G>T** |  |  |  |
| AA + TT | 7 | 1.84 ± 7.78 | 0.98 |
| AC or CC + GT or GG | 48 | 1.74 ± 25.47 |  |
| AA or AC + TT or GT | 65 | 5.19 ± 18.36 | 0.34 |
| CC + GG | 4 | -9.63 ± 25.81 |  |
| ***XPF* c.2505T>C *+ TP53* c.215G>C** |  |  |  |
| TT + CC | 52 | 2.61 ± 21.37 | 0.09 |
| TC or CC + GC or GG | 6 | 18.17 ± 17.89 |  |
| TT or TC + GC or CC |  | NE |  |
| CC + GG |  |  |  |
| ***XPF* c.2505T>C *+ FAS* c.-671A>G** |  |  |  |
| TT + GG | 12 | 2.20 ± 17.01 | 0.98 |
| TC or CC + AG or AA | 43 | 2.32 ± 18.71 |  |
| TT or TC + GG or AG |  | NE |  |
| CC + AA |  |  |  |
| ***XPF* c.2505T>C *+ FAS* c.-1378G>A** |  |  |  |
| TT + AA | 3 | 9.21 ± 20.59 | 0.78 |
| TC or CC + GA or GG | 53 | 3.65 ± 19.02 |  |
| TT or TC + AA or GA | 27 | 0.31 ± 21.92 | 0.82 |
| CC + GG | 6 | -3.14 ± 34.18 |  |
| ***XPF* c.2505T>C + *FASL* c.-844C>T** |  |  |  |
| TT + TT | 14 | 5.59 ± 15.85 | 0.96 |
| TC or CC + CT or CC | 44 | 5.35 ± 18.50 |  |
| TT or TC + TT or CT | 72 | 4.01 ± 17.62 | 1.00 |
| CC or CC | 3 | 4.18 ± 41.16 |  |
| ***XPF* c.2505T>C + *CASP3* c.-1191A>G** |  |  |  |
| TT + GG | 6 | 6.57 ± 14.24 | 0.59 |
| TC or CC + AG or AA | 52 | 2.93 ± 21.46 |  |
| TT or TC + GG or AG | 60 | 3.20 ± 18.27 | 0.65 |
| CC + AA | 4 | -8.04 ± 44.10 |  |
| ***XPF* c.2505T>C *+ CASP3* c.-182-247G>T** |  |  |  |
| TT + TT | 6 | 1.83 ± 6.88 | 0.78 |
| TC or CC + GT or GG | 50 | 2.99 ± 21.60 |  |
| TT or TC + TT or GT | 67 | 4.95 ± 17.23 | 0.87 |
| CC + GG | 5 | 2.12 ± 36.95 |  |
| ***ERCC1* c.354C>T *+ TP53* c.215G>C** |  |  |  |
| CC + CC | 5 | 6.70 ± 15.34 | 0.83 |
| CT or TT + GC or GG | 75 | 5.03 ± 17.01 |  |
| CC or CT + CC or GC | 50 | 2.79 ± 22.77 | 0.38 |
| TT + GG | 12 | 8.58 ± 19.35 |  |
| ***ERCC1* c.354C>T *+ FAS* c.-671A>G** |  |  |  |
| CC + GG | 7 | 5.40 ± 24.97 | 0.88 |
| CT or TT+ AG or AA | 62 | 6.91 ± 15.56 |  |
| CC or CT + GG or AG | 60 | 3.09 ± 20.85 | 0.65 |
| TT + AA | 5 | 7.45 ± 19.54 |  |
| ***ERCC1* c.354C>T *+ FAS* c.-1378G>A** |  |  |  |
| CC + AA |  | NE |  |
| CT or TT + GA or GG |  |  |  |
| CC or CT+ AA or GA | 25 | 0.08 ± 23.74 | 0.30 |
| TT + GG | 16 | 6.39 ± 14.53 |  |
| ***ERCC1* c.354C>T *+ FASL* c.-844C>T** |  |  |  |
| CC + TT | 6 | -7.30 ± 24.07 | 0.20 |
| CT or TT+ CT or CC | 60 | 7.18 ± 15.66 |  |
| CC or CT+ TT or CT | 66 | 2.80 ± 19.32 | 0.84 |
| TT + CC | 9 | 3.93 ± 14.98 |  |
| ***ERCC1* c.354C>T *+ CASP3* c.-1191A>G** |  |  |  |
| CC + GG | 5 | 3.48 ± 10.72 | 0.65 |
| CT or TT+ AG or AA | 75 | 5.96 ± 17.47 |  |
| CC or CT+ GG or AG | 55 | 2.94 ± 19.56 | 0.64 |
| TT + AA | 11 | 5.92 ± 18.56 |  |
| ***ERCC1* c.354C>T *+ CASP3* c.-182-247G>T** |  |  |  |
| CC + TT | 3 | -2.67 ± 3.94 | 0.02 |
| CT or TT+ GT or GG | 71 | 6.30 ± 17.88 |  |
| CC or CT+ TT or GT | 55 | 4.31 ± 18.59 | 0.44 |
| TT + GG | 5 | 8.69 ± 10.59 |  |
| ***MLH1* c.93G>A *+ TP53* c.215G>C** |  |  |  |
| GG + CC | 6 | 4.01 ± 12.70 | 0.95 |
| GA or AA + GC or GG | 41 | 3.59 ± 19.74 |  |
| GG or GA + CC or GC |  | NE |  |
| AA + GG |  |  |  |
| ***MLH1* c.93G>A *+ FAS* c.-671A>G** |  |  |  |
| GG + GG | 15 | 5.65 ± 18.67 | 0.78 |
| GA or AA + AG or AA | 35 | 7.26 ± 17.45 |  |
| GG or GA + GG or AG |  | NE |  |
| AA + AA |  |  |  |
| ***MLH1* c.93G>A *+ FAS* c.-1378G>A** |  |  |  |
| GG + AA | 5 | 11.12 ± 24.31 | 0.74 |
| AG or AA + GA or GG | 44 | 7.22 ± 17.54 |  |
| GG or AG + AA or GA | 27 | -0.18 ± 22.04 | 0.02 |
| AA + GG | 2 | 22.94 ± 6.25 |  |
| ***MLH1* c.93G>A *+ FASL* c.-844C>T** |  |  |  |
| GG + TT | 14 | 1.92 ± 20.96 | 0.32 |
| AG or AA + CT or TT | 33 | 8.50 ± 18.37 |  |
| GG or AG + TT or CT |  | NE |  |
| AA + CC |  |  |  |
| ***MLH1* c.93G>A *+ CASP3* c.-1191A>G** |  |  |  |
| GG + GG | 5 | 1.03 ± 9.44 | 0.44 |
| GA or AA + AG or AA | 40 | 5.35 ± 20.89 |  |
| GG or AG or GG or AG | 62 | 3.66 ± 18.97 | 0.21 |
| AA + AA | 2 | 40.54 ± 18.64 |  |
| ***MLH1* c.93G>A *+ CASP3* c.-182-247G>T** |  |  |  |
| GG + TT | 6 | 3.12 ± 14.27 | 0.63 |
| GA or AA + GT or TT | 39 | 6.50 ± 21.54 |  |
| GG or AG + TT or GT | 68 | 4.38 ± 18.23 | 0.54 |
| AA + GG | 2 | 27.52 ± 37.05 |  |
| ***MSH2* c.211+9C>G *+ TP53* c.215G>C** |  |  |  |
| CC + CC | 3 | 16.42 ± 25.94 | 0.50 |
| CG or GG + GC or GG | 76 | 4.27 ± 18.61 |  |
| CC or CG + CC or GC | 41 | 4.22 ± 23.36 | 0.83 |
| GG + GG | 10 | 2.48 ± 22.95 |  |
| ***MSH2* c.211+9C>G *+ FAS* c.-671A>G** |  |  |  |
| CC + GG | 4 | -13.08 ± 35.89 | 0.40 |
| CG or GG + AG or AA | 62 | 4.34 ± 18.19 |  |
| CC or CG + AG or GG | 55 | 4.53 ± 20.70 | 0.52 |
| GG + AA | 7 | -1.43 ± 21.93 |  |
| ***MSH2* c.211+9C>G *+ FAS* c.-1378G>A** |  |  |  |
| CC + AA | 3 | -14.44 ± 47.13 | 0.56 |
| CG or GG + GA or GG | 80 | 4.64 ± 18.09 |  |
| CC or CG + AA or GA | 24 | 4.24 ± 23.57 | 0.98 |
| GG + GG | 22 | 4.11 ± 17.18 |  |
| ***MSH2* c.211+9C>G *+ FASL* c.-844C>T** |  |  |  |
| CC + TT | 8 | -0.82 ± 30.22 | 0.54 |
| GC or GG + CT or TT | 65 | 6.14 ± 18.26 |  |
| CC or GC + TT or CT | 55 | 3.91 ± 19.96 | 0.29 |
| GG + CC | 5 | -9.03 ± 23.20 |  |
| ***MSH2* c.211+9C>G *+ CASP3* c.-1191A>G** |  |  |  |
| CC + GG | 5 | -0.25 ± 12.20 | 0.44 |
| CG or GG + AG or AA | 78 | 4.61 ± 18.63 |  |
| CC or CG + GG or AG | 52 | 4.72 ± 18.88 | 0.69 |
| GG + AA | 15 | 2.49 ± 18.88 |  |
| ***MSH2* c.211+9C>G *+ CASP3* c.-182-247G>T** |  |  |  |
| CC + TT | 2 | 3.00 ± 4.24 | 0.58 |
| CG or GG + GT or TT | 73 | 5.38 ± 19.04 |  |
| CC or CG + TT or GT | 52 | 5.49 ± 18.68 | 0.33 |
| GG + GG | 9 | -2.13 ± 21.02 |  |
| ***MSH3* c.3133A>G *+ TP53* c.215G>C** |  |  |  |
| AA + CC | 3 | 5.51 ± 15.80 | 0.87 |
| AG or GG + GC or GG | 40 | 3.76 ± 18.80 |  |
| AA or AG + CC or GC | 54 | 2.79 ± 21.75 | 0.44 |
| GG + GG | 6 | 10.01 ± 20.46 |  |
| ***MSH3* c.3133A>G *+ FAS* c.-671G>A** |  |  |  |
| AA + GG | 17 | -2.21 ± 24.50 | 0.35 |
| AG or GG + AG or AA | 39 | 3.65 ± 18.52 |  |
| AA or AG + GG or AG |  | NE |  |
| GG + AA |  |  |  |
| ***MSH3* c.3133A>G *+ FAS* c.-1378G>A** |  |  |  |
| AA +AA | 5 | -8.97 ± 34.16 | 0.42 |
| AG or GG + GA or GG | 46 | 4.96 ± 18.27 |  |
| AA or AG + AA or GA | 26 | 1.27 ± 24.50 | 0.30 |
| GG +GG | 7 | 11.30 ± 20.27 |  |
| ***MSH3* c.3133A>G *+ FASL* c.-844C>T** |  |  |  |
| AA + TT | 15 | -3.30 ± 26.66 | 0.24 |
| AG or GG + CT or CC | 36 | 5.97 ± 20.56 |  |
| AA or AG + TT or CT | 69 | 3.67 ± 19.72 | 0.55 |
| GG + CC | 2 | 25.20 ± 35.64 |  |
| ***MSH3* c.3133A>G *+ CASP3* c.-1191A>G** |  |  |  |
| AA + GG | 8 | 1.56 ± 10.54 | 0.45 |
| AG or GG + AG or AA | 45 | 5.16 ± 19.03 |  |
| AA or AG + GG or AG | 56 | 3.04 ± 18.86 | 0.76 |
| GG + AA | 2 | 9.90 ± 24.17 |  |
| ***MSH3* c.3133A>GG *+ CASP3* c.-182-247G>T** |  |  |  |
| AA + TT | 5 | 2.67 ± 15.83 | 0.64 |
| AG or GG + GT or GG | 40 | 6.46 ± 20.32 |  |
| AA or AG + TT or GT |  | NE |  |
| GG + GG |  |  |  |
| ***EXO1* c.1762G>A *+ TP53* c.215G>C** |  |  |  |
| GG + CC | 4 | 25.14 ± 18.11 | 0.07 |
| GA or AA + GC or GG | 57 | 0.85 ± 19.60 |  |
| GG or AG + CC or GC | 52 | 2.87 ± 22.25 | 0.74 |
| AA + GG | 5 | -1.22 ± 24.97 |  |
| ***EXO1* c.1762G>A *+ FAS* c.-671A>G** |  |  |  |
| GG + GG | 14 | 1.22 ± 25.93 | 0.89 |
| GA or AA + AG or AA | 52 | 0.22 ± 19.00 |  |
| GG or GA + GG or AG |  | NE |  |
| AA + AA |  |  |  |
| ***EXO1* c.1765G>A *+ FAS* c.-1378G>A** |  |  |  |
| GG + AA | 5 | -8.97 ± 34.16 | 0.56 |
| GA or AA + GA or GG | 62 | 0.68 ± 18.29 |  |
| GG or GA + AA or GA | 27 | 2.24 ± 24.21 | 0.77 |
| AA + GG | 9 | 4.67 ± 19.65 |  |
| ***EXO1* c.1765G>A *+ FASL* c.-844C>T** |  |  |  |
| GG + TT | 14 | 1.53 ± 24.09 | 0.95 |
| AG or AA + CT or CC | 51 | 1.96 ± 18.77 |  |
| GG or GA + TT or CT | 70 | 3.44 ± 19.09 | 0.23 |
| AA + CC | 4 | -6.78 ± 13.67 |  |
| ***EXO1* c.1762G>A *+ CASP3* c.-1191A>G** |  |  |  |
| GG + GG | 5 | 10.12 ± 14.62 | 0.27 |
| GA or AA + AG or AA | 58 | 1.46 ± 19.59 |  |
| GG or AG + GG or AG | 59 | 4.04 ± 19.02 | 0.99 |
| AA + AA | 6 | 3.89 ± 20.78 |  |
| ***EXO1* c.1762G>A *+ CASP3* c.-182-247G>T** |  |  |  |
| GG + TT | 4 | 8.10 ± 6.38 | 0.16 |
| AG or AA + GT or GG | 55 | 1.67 ± 19.84 |  |
| GG or AG + TT or GT | 64 | 4.92 ± 17.79 | 0.33 |
| AA + GG | 5 | 0.58 ± 7.93 |  |
| ***Combined SNVs in intrinsic/extrinsic apoptosis genes*** | | | |
| ***TP53* c.215G>C *+ FAS* c.-671A>G** |  |  |  |
| GG + AA | 17 | 2.79 ± 18.77 | 0.89 |
| GC or CC + AG or GG | 41 | 3.55 ± 21.41 |  |
| GG or GC + AA or AG | 75 | 2.91 ± 19.07 | 0.27 |
| CC + GG | 3 | 14.88 ± 14.04 |  |
| ***TP53* c.215G>C *+ FAS* c.-1378G>A** |  |  |  |
| GG + GG | 38 | 4.89 ± 16.86 | 0.71 |
| GC or CC + GA or AA | 16 | 2.23 ± 25.45 |  |
| GG or GC + GG or GA |  | NE |  |
| CC + AA |  |  |  |
| ***TP53* c.215G>C *+ FASL* c.-844C>T** |  |  |  |
| GG + CC | 13 | 6.97 ± 20.92 | 0.76 |
| GC or CC + CT or TT | 39 | 4.87 ± 20.82 |  |
| GG or GC + CC or CT | 78 | 4.15 ± 19.11 | 0.47 |
| CC + TT | 7 | 9.37 ± 17.09 |  |
| ***TP53* c.215G>C *+ CASP3* c.-1191A>G** |  |  |  |
| GG + GG | 19 | 2.11 ± 22.67 | 0.93 |
| GC or CC + GA or AA | 32 | 2.71 ± 22.06 |  |
| GG or GC + GG or GA |  | NE |  |
| CC +AA |  |  |  |
| ***TP53* c.215G>C *+ CASP3* c.-182-247G>T** |  |  |  |
| GG + GG | 18 | 2.27 ± 17.01 | 0.59 |
| GC or CC + GT or TT | 37 | 4.98 ± 17.96 |  |
| GG or GC + GG or GT | 87 | 2.80 ± 20.80 | 0.93 |
| CC + TT | 2 | 2.41 ± 4.54 |  |
| ***FAS* c.-671A>G + *FAS* c.-1378G>A** |  |  |  |
| AA + GG | 29 | 2.10 ± 20.80 | 0.85 |
| AG or GG + GA or AA | 24 | 0.92 ± 24.85 |  |
| AA or AG + GG or GA | 78 | 3.08 ± 18.83 | 0.39 |
| GG + AA | 2 | -36.26 ± 39.81 |  |
| ***FAS* c.-671A>G + *FASL* c.-844C>T** |  |  |  |
| AA + CC | 9 | 0.24 ± 24.64 | 0.67 |
| AG or GG + CT or TT | 52 | 4.09 ± 19.36 |  |
| AA or AG + CC or CT | 64 | 3.79 ± 19.21 | 0.27 |
| GG + TT | 8 | -7.75 ± 26.70 |  |
| ***FAS* c.-671A>G *+ CASP3* c.-1191A>G** |  |  |  |
| AA + AA | 13 | -4.37 ± 22.18 | 0.34 |
| AG or GG + AG or GG | 43 | 2.36 ± 19.21 |  |
| AA or AG + AA or AG |  | NE |  |
| GG + GG |  |  |  |
| ***FAS* c.-671A>G *+ CASP3* c.-182-247G>T** |  |  |  |
| AA + GG | 11 | -1.37 ± 30.43 | 0.51 |
| AG or GG + GT or TT | 47 | 5.11 ± 20.42 |  |
| AA or AG + GG or GT |  | NE |  |
| GG + TT |  |  |  |
| ***FAS* c.-1378G>A *+ FASL* c.-844C>T** |  |  |  |
| GG + CC | 25 | 2.84 ± 23.85 | 0.86 |
| GA or AA + CT or TT | 22 | 1.52 ± 26.02 |  |
| GG or GA + CC or CT | 77 | 4.70 ± 19.09 | 0.72 |
| AA + TT | 2 | -17.60 ± 66.21 |  |
| ***FAS* c.-1378G>A *+ CASP3* c.-1191A>G** |  |  |  |
| GG + AA | 33 | 1.92 ± 20.23 | 0.59 |
| GA or AA + AG or GG | 17 | -1.57 ± 22.43 |  |
| GG or GA + AA or AG |  | NE |  |
| AA + GG |  |  |  |
| ***FAS* c.-1378G>A *+ CASP3* c.-182-247G>T** |  |  |  |
| GG + GG | 26 | -0.08 ± 18.92 | 0.95 |
| GA or AA + GT or TT | 16 | 0.29 ± 18.90 |  |
| GG or GA + GG or GT |  | NE |  |
| AA + TT |  |  |  |
| ***FASL* c.-844C>T *+ CASP3* c.-1191A>G** |  |  |  |
| CC + AA | 11 | 0.87 ± 18.89 | 0.63 |
| CT or TT + AG or GG | 43 | 3.92 ± 16.14 |  |
| CC or CT + AA or AG | 75 | 4.96 ± 19.85 | 0.86 |
| TT + GG | 4 | 6.93 ± 19.59 |  |
| ***FASL* c.-844C>T *+ CASP3* c.-182-247G>T** |  |  |  |
| CC + GG | 11 | 3.68 ± 21.10 | 0.73 |
| CT or TT + GT or TT | 49 | 6.05 ± 16.05 |  |
| CC or CT + GG or GT | 72 | 4.95 ± 20.04 | 0.21 |
| TT + TT | 3 | -4.28 ± 9.17 |  |
| ***CASP3* c.-1191A>G *+ CASP3* c.-182-247G>T** |  |  |  |
| AA + GG | 11 | -0.74 ± 26.62 | 0.48 |
| AG or GG + GT or TT | 36 | 5.43 ± 16.95 |  |
| AA or AG + GG or GT |  | NE |  |
| GG + TT |  |  |  |

N: number of patients. eGFR: estimated glomerular filtration rate. Δ eGFR represents
pre-chemotherapy value - post chemotherapy value divided by the pre-chemotherapy value. NER: nucleotide excision repair pathway. MMR: mismatch repair pathway. Results with significant *P-*values (< 0.10) and more than 10 individuals in each group are presented in bold letters. Note: The number of patients in each genotype combination may vary because only individuals with both SNVs available were included in the analysis.
